# Strategic Combinations of Antibody–Drug Conjugates from 2023 to 2025: From Dual Therapies to Innovative ADC-Based Regimens

**DOI:** 10.3390/pharmaceutics17121581

**Published:** 2025-12-08

**Authors:** Heewon Jang, Ji-Eun Chang

**Affiliations:** College of Pharmacy, Dongduk Women’s University, Seoul 02748, Republic of Korea

**Keywords:** antibody–drug conjugates, combination therapy, cancer treatment, multiplet regimens, therapeutic optimization

## Abstract

Antibody–drug conjugates (ADCs) are a potent class of targeted cancer therapies that combine the specificity of monoclonal antibodies with the cytotoxic potency of chemotherapeutic agents. By targeting tumor cells with minimal impact on healthy tissues, ADCs achieve a favorable balance between efficacy and systemic toxicity. This therapeutic modality has demonstrated significant clinical success, as evidenced by the FDA approval of 15 ADCs by 2025, with one later withdrawn due to safety concerns, and indications continuing to expand across various cancer types. Beyond monotherapy, there is growing interest in ADC-based combination strategies aimed at enhancing therapeutic outcomes and managing resistance. Several combinations, especially with chemotherapy, immune checkpoint inhibitors, or molecularly targeted agents, have gained regulatory approval or advanced to late-stage clinical trials. While dual-agent regimens have historically dominated the research landscape, multiplet combinations are also gaining traction and represent a promising frontier in oncology. This evolving paradigm highlights the need for a comprehensive understanding of current ADC combination approaches. In this review, we examine recent clinical advances in ADC-based combinations, with a particular focus on regimens that incorporate FDA-approved ADCs. We also discuss the progression from dual-agent approaches to more complex multiplet strategies across a range of tumor types.

## 1. Introduction

Antibody–drug conjugates (ADCs) represent a next-generation modality in targeted cancer therapy that consist of a monoclonal antibody (mAb) chemically linked to a highly cytotoxic small-molecule payload via a stable linker. This structural design enables ADCs to selectively deliver cytotoxic agents to tumor cells by exploiting the high antigen specificity of the antibody, while minimizing off-target toxicity to normal tissues. Through this mechanism, ADCs enhance therapeutic efficacy and safety compared to conventional chemotherapy, which often causes substantial damage to healthy tissues [1,2,3,4].

An ADC is composed of three essential components: the antibody, the linker, and the payload. The antibody is typically based on an IgG1 backbone and targets antigens that are selectively overexpressed in tumor cells or within the tumor microenvironment. Ideally, the antibody should demonstrate high specificity and affinity, with minimal cross-reactivity to normal tissues, and low immunogenicity. For this reason, antibody engineering has progressed from chimeric to humanized, and now to fully human antibodies. The linker connects the antibody to the cytotoxic payload and must be stable in circulation to prevent premature drug release, while also being cleavable or activatable under tumor-specific conditions to ensure efficient payload delivery within the tumor cell. Cleavable linkers respond to factors such as acidic pH, reductive environments, or specific enzymatic activity, whereas non-cleavable linkers release the payload only after complete intracellular degradation of the antibody. The payload is the cytotoxic component responsible for the antitumor activity of the ADC. It must be potent enough to kill cancer cells at low concentrations while maintaining selectivity to minimize systemic toxicity. Commonly used payloads include tubulin inhibitors, DNA-damaging agents, and topoisomerase I inhibitors. Thus, the therapeutic efficacy and safety of ADCs depend heavily on the careful selection and integration of each component [1,2,3,4].

The mechanism of action of ADCs can be summarized in five key steps: antigen binding, internalization, lysosomal degradation, and payload release followed by cell death. The antibody binds to a specific antigen on the tumor cell surface and is internalized via receptor-mediated endocytosis. After trafficking through endosomes to the lysosome, the antibody and linker are degraded, releasing the cytotoxic payload. The released payload exerts its effect by inhibiting cell division or inducing DNA damage, leading to tumor cell death. Some ADCs also exhibit a bystander effect, wherein the released payload diffuses into the tumor microenvironment and kills neighboring tumor cells regardless of antigen expression [3,4].

Up to now, the U.S. Food and Drug Administration (FDA) has approved a total of 15 ADCs. In 2025, two new ADCs—datopotamab deruxtecan and telisotuzumab vedotin (Teliso-V)—received regulatory approval. Datopotamab deruxtecan (Dato-DXd) was granted full approval on 17 January 2025, for the treatment of patients with hormone receptor-positive, HER2-negative metastatic breast cancer who had previously received endocrine therapy and chemotherapy, based on the phase III TROPION-Breast01 trial. In this study, Dato-DXd significantly prolonged progression-free survival (PFS) compared to chemotherapy, with median progression free survival (mPFS) of 6.9 months vs. 4.9 months. Subsequently, on 23 June 2025, it received accelerated approval for EGFR-mutant non-small-cell lung cancer (NSCLC) based on findings from the TROPION-Lung05 and TROPION-Lung01 studies [5,6]. Meanwhile, Teliso-V received accelerated approval on 14 May 2025, for patients with non-squamous NSCLC exhibiting high c-Met protein overexpression. This decision was based on results from the phase II LUMINOSITY trial, which reported an objective response rate (ORR) of 35% and a median duration of response (DOR) of 7.2 months [7]. Table 1 summarizes the FDA-approved ADCs as of August 2025.

ADCs have shown clinical success as monotherapy in various cancers, and combination strategies are being increasingly investigated to overcome resistance, improve efficacy, and enhance safety. The rationale for such combinations lies in the complementary mechanisms between ADCs and other therapeutic agents. Combining ADCs with conventional chemotherapeutic agents, including microtubule inhibitors, anthracyclines, antimetabolites, alkylating agents, and topoisomerase inhibitors, can produce synergistic effects by targeting different phases of the cell cycle. In some cases, using ADCs allows for dose reductions in chemotherapy, which may minimize treatment-related toxicity while maintaining or even improving antitumor effects. Immune checkpoint inhibitors (ICIs) have become central to cancer immunotherapy, yet many patients do not experience long-lasting responses with ICI monotherapy. ADCs can induce immunogenic cell death, remodel the tumor microenvironment, and promote T-cell activation, thereby creating a more favorable setting for ICIs to exert their effects. Moreover, combining ADCs with molecular targeted therapies, such as tyrosine kinase inhibitors, monoclonal antibodies, and T-cell engaging bispecific antibodies, enables a more precise treatment approach. These combinations may help overcome resistance mechanisms such as decreased antigen expression or alternative signaling activation, and offer safer, more effective options for older or clinically fragile patients. Together, these approaches support the growing interest in ADC-based combination therapies as a means to expand treatment possibilities and improve clinical outcomes across multiple cancer types.

## 2. Combination Strategies with ADCs (2023–2025)

### 2.1. Recent Dual Combination Strategies

#### 2.1.1. ADCs Combined with Chemotherapy

Recently, ADCs have been increasingly combined with conventional chemotherapy to maximize therapeutic efficacy. Since ADCs and chemotherapeutic agents act on different phases of the cell cycle, their combination is expected to produce complementary antitumor effects [2]. In some cases, replacing part of the standard chemotherapy with an ADC may reduce treatment-related toxicity, while in relapsed or refractory cancers, ADCs can enhance efficacy without increasing chemotherapy intensity [16,40,41,42]. Moreover, in tumors with high target antigen expression, ADCs may further augment the antitumor response [42,43]. ADC-based combinations are also being explored as first-line treatment options for older or vulnerable patients and as a means to achieve deeper responses for long-term remission and relapse prevention [40,44,45]. As a result, a growing number of clinical trials are actively investigating ADC-chemotherapy combinations across a wide range of cancer types, reflecting significant clinical interest in this approach. Table 2 summarizes the clinical trials on ADC-chemotherapy combination regimens.

***Gemtuzumab ozogamicin***

Gemtuzumab ozogamicin (GO) is an ADC targeting the CD33 antigen and is approved for the treatment of acute myeloid leukemia (AML). Recent studies have demonstrated that GO continues to play an important role in AML therapy, with active efforts aimed at maximizing its efficacy in specific molecular subtypes.

For example, in the phase III AMLSG 09–09 trial involving patients with NPM1-mutated AML, the addition of GO significantly reduced relapse rates and achieved superior clearance of NPM1 transcripts. The results from this study provide further evidence that GO should be added to the standard of care treatment in adults with NPM1-mutated AML [46]. Similarly, in the NCRI AML19 trial, the addition of GO to the fludarabine, cytarabine, granulocyte colony-stimulating factor, and idarubicin regimen (FLAG-Ida) significantly improved event-free survival (EFS) and reduced relapse rates, while also providing substantial overall survival (OS) benefits in patients with NPM1- and FLT3-mutated AML. These findings strongly suggest that GO is particularly effective in patient subsets harboring specific genetic mutations [47]. Together, these results indicate that GO is particularly effective in genetically defined subgroups, reinforcing the rationale for its use in mutation-specific treatment strategies. Building on this rationale, a recent phase I/Ib study evaluated the combination of CPX-351, a liposomal formulation of daunorubicin and cytarabine, with GO in newly diagnosed, CD33-positive AML. The combination yielded high complete remission and complete remission with incomplete hematologic recovery rates, with MRD negativity observed in nearly all responders in preliminary analyses [43].

These benefits were achieved while maintaining a manageable safety profile, with hematologic toxicities and occasional hepatic events occurring at expected rates and generally controlled through appropriate monitoring.

***Brentuximab vedotin***

Brentuximab vedotin (BV) is an anti-CD30 ADC that was initially approved as monotherapy for relapsed classical Hodgkin lymphoma (cHL). BV-based combinations show high efficacy and tolerability across lymphoma subtypes, with several approvals by FDA underscoring their potential as standard therapies. In a multicenter phase II study in patients with CD30-positive peripheral T-cell lymphoma (PTCL), induction therapy with BV plus cyclophosphamide, doxorubicin, etoposide, prednisone (CHEP–BV) followed by BV consolidation achieved a 2-year PFS rate of 59% and an OS rate of 86%, representing the first clinical evidence for the efficacy of BV in combination with etoposide [48]. In the SGN35-015 trial involving cHL patients aged ≥60 years ineligible for standard chemotherapy, BV plus dacarbazine (BV–DTIC) yielded a median duration of response (mDOR) of 46.0 months, outperforming BV monotherapy while maintaining a favorable safety profile [44]. To mitigate the toxicity associated with BV plus doxorubicin, vinblastine, and dacarbazine (BV–AVD), a phase II trial evaluated BV plus doxorubicin and dacarbazine (BV–AD), omitting vinblastine and bleomycin, and reported markedly reduced rates of peripheral neuropathy alongside an ORR of 100% and a CR rate of 97% [41]. In patients with extranodal NK/T-cell lymphoma (ENKTL), the B-MAD regimen, combining BV with methotrexate, L-asparaginase, and dexamethasone, achieved a CR rate of 66.7% with good tolerability [49]. Meanwhile, the GELTAMO trial in treatment-naïve elderly cHL patients evaluated BV in combination with cyclophosphamide, procarbazine, prednisone, etoposide, and mitoxantrone (BrEPEM), achieving a 93% ORR in the per-protocol analysis; although grade ≥ 3 neutropenia was observed in some patients, the findings suggest that with appropriate prophylactic measures, the clinical benefits of BV-containing regimens can be maintained [40].

Notably, neuropathy—BV’s characteristic toxicity—was observed but was typically reversible or manageable with dose adjustments, allowing most patients to continue treatment successfully.

***Inotuzumab ozogamicin***

Inotuzumab ozogamicin (InO) is a CD22-targeted ADC that has shown consistent efficacy and tolerability when combined with chemotherapy in CD22-positive B-cell precursor acute lymphoblastic leukemia (B-ALL), regardless of patient age, disease status, or treatment history. Combination strategies with agents of different mechanisms are actively being explored to reduce the toxicity and relapse risk of intensive chemotherapy and improve long-term outcomes. In the EWALL-INO phase II study in elderly patients with Philadelphia chromosome-negative B-ALL (Ph– B-ALL), first induction therapy with vincristine, dexamethasone, and InO, followed by a second induction with cyclophosphamide, dexamethasone, and InO, resulted in a 1-year OS rate of 73.2% and a relapse-free survival (RFS) rate of 66%. Notably, a complete remission or complete remission with incomplete platelet recovery was achieved in 90% of patients after the second induction [45]. The ITCC-059 phase Ib trial in pediatric patients with relapsed/refractory (R/R) CD22+ B-ALL evaluated InO in combination with vincristine, dexamethasone, and intrathecal therapy, yielding an ORR of 80% and a MRD negativity rate of 66.7%, with an established safety profile at 1.8 mg/m^2^ per cycle [16]. In adult R/R B-ALL, the phase I dose-escalation study of the dose-optimized etoposide, prednisone, vincristine, cyclophosphamide, and doxorubicin (DA-EPOCH) plus InO achieved a morphologic CR rate of 84% and an ORR of 83% in patients, while serious hepatotoxicities, including sinusoidal obstruction syndrome (SOS), were infrequent [50]. Furthermore, the INITIAL-1 phase II trial in elderly patients with Ph- B-ALL reported that all 43 evaluable patients experienced complete remission, with or without complete hematologic recovery following induction therapy consisting of InO in combination with dexamethasone, low-dose cyclophosphamide, and intrathecal methotrexate/cytarabine/dexamethasone. Severe treatment-related adverse events were uncommon, suggesting that this approach may help preserve quality of life. Collectively, these findings underscore that InO combined with chemotherapy represents a potent and well-tolerated therapeutic approach applicable across a broad spectrum of ages and treatment settings, from frontline to R/R disease [51].

While these regimens were generally well tolerated, hepatotoxicity-including the risk of veno-occlusive disease (VOD)-remained an important consideration, particularly for patients undergoing subsequent stem-cell transplantation.

***Tisotumab vedotin***

Tisotumab vedotin (TV) is an ADC targeting tissue factor (TF), a protein highly expressed in multiple solid tumors including cervical cancer. Currently approved as monotherapy, TV is now being actively investigated in combination regimens for recurrent or metastatic cervical cancer (r/mCC). The innovaTV 205/GOG-3024/ENGOT-cx8 phase Ib/II multicenter trial evaluated TV in combination with carboplatin in patients with r/mCC. Using a dose-escalation followed by a dose-expansion design, the regimen achieved an ORR of 54.5% and a mDOR of 8.6 months, with a manageable safety profile. These findings indicate that TV plus carboplatin is well tolerated and provides durable antitumor activity, supporting further exploration of this combination in the treatment of r/mCC [42].

***Mirvetuximab soravtansine***

Mirvetuximab soravtansine (MIRV) is an ADC targeting folate receptor alpha (FRα) that has shown promising efficacy as monotherapy in FRα-positive, platinum-resistant epithelial ovarian cancer (EOC), leading to its accelerated FDA approval. This phase I study investigated MIRV in combination with gemcitabine in patients with FRα-positive recurrent EOC, primary peritoneal cancer, fallopian tube cancer, endometrial cancer, or triple-negative breast cancer, with the aim of defining a tolerable dosing regimen and assessing safety. Across all enrolled patients, the combination achieved a confirmed partial response (PR) rate of 15%, showing promising activity in platinum-resistant epithelial ovarian cancer patients treated at the recommended phase II dose (RP2D) compared with other cancer types. Although frequent hematologic toxicities were reported and the response rate was lower than in MIRV monotherapy trials, the study provides meaningful insights into the potential of combining targeted therapy with cytotoxic chemotherapy [52]. The regimen’s safety profile was consistent with known MIRV-associated ocular and hematologic toxicities, most of which were manageable with appropriate dose modifications.

**Table 2:** Summary of clinical trials on ADC-chemotherapy combination regimens. This table provides an overview of partner drugs, indications, primary efficacy and safety outcomes, clinical trial details, and publication year.

ADC	Target	Partner Drug	Indication	Primary Endpoint		Phase	NCT Number	Publication Year	Ref.
				Efficacy	Safety				
Gemtuzumab Ozogamicin	CD33	Idarubicin, Cytarabine, Etoposide, ATRA	NPM1-mutated AML	6 months EFS 58%, OS 73%		Phase III	NCT00893399	2023	[43]
		CPX-351	Newly diagnosed AML		MTD: Immature Grade ≥ 3 AEs cytopenias 100%, febrile neutropenia 67%, thrombocytopenia 22%	Phase I	NCT05558124	2024	[46]
		FLAG-Ida vs. DA	Younger patients with newly diagnosed AML	OS 66% vs. 63% NPM1-mutated 82% vs. 64% FLT3-mutated 64% vs. 54%		-	ISRCTN78449203	2024	[47]
Brentuximab Vedotin	CD30	Doxorubicin, Dacarbazine	Non bulky limited-stage cHL	CR rate 97%		Phase II	NCT02505269	2023	[41]
		B-MAD	Extranodal NK/T-cell lymphoma		RP2D BV 1.8 mg/kg Grade ≥ 3 AEs anemia 16.7%, leukopenia 29.2%, neutropenia 29.2%, and elevated ALT 4.2%	Phase I Phase II	NCT03246750	2023	[49]
		Cyclophosphamide, Doxorubicin, Etoposide, Prednisone	CD30+ peripheral T-cell lymphomas	CR rate 79%		Phase II	NCT03113500	2024	[48]
		Dacarbazine	Advanced-stage cHL in patients aged ≥60 years who are unfit for conventional chemotherapy	ORR 95%		Phase II	NCT01716806	2024	[44]
		Cyclophosphamide, Procarbazine, Prednisone, Etoposide, Mitoxantrone	Older patients wiith untreated cHL	CR rate 89%	no DLTs MTD BV 1.2 mg/kg Grade ≥ 3 AEs 17% neutropenia 7%, anemia 3%	Phase Ib Phase II	NCT03576378	2025	[40]
Inotuzumab Ozogamicin	CD22	Dexamethasone, Methotrexate, Cytarabine, Mercaptopurine	Older patients with Ph- B-precursor ALL	1-year EFS 88%		Phase II	NCT03460522	2023	[51]
		First induction therapy: Vincristine, Dexamethasone Second induction therapy: Cyclophosphamide, Dexamethasone	Ph- CD22+ B-cell precursor ALL	1-year OS 73.2%		Phase II	NCT03249870	2024	[45]
		Vincristine, Dexamethasone	Pediatric B-cell precursor CD22+ ALL		RP2D InO 1.8 mg/m^2^/cycle	Phase Ib	NTR5736	2024	[16]
		DA-EPOCH	Adults with R/R B-cell ALL		MTD InO 0.6 mg/m^2^ on days 8 and 15	Phase I	NCT03991884	2024	[50]
Tisotumab Vedotin	TF	Carboplatin	Recurrent or metastatic cervical cancer	dose-expansion: ORR 54.5%	dose-escalation: DLTs 0%, RP2D TV 2 mg/kg/cycle	Phase I Phase II	NCT03786081	2023	[42]
Mirvetuximab Soravtansine	FRα	Gemcitabine	FRα-positive recurrent ovarian, primary peritoneal, fallopian tube, or endometrial cancer, or TNBC		MTD, RP2D MIRV 6 mg/kg on day 1, Gemcitabine 800 mg/m^2^ on days 1 and 8	Phase I	NCT02996825	2024	[52]

AEs: adverse events, ALL: acute lymphoblastic leukemia, AML: acute myeloid leukemia, ATRA: all-trans retinoic acid, B-MAD: methotrexate, L-asparaginase, dexamethasone, BV: brentuximab vedotin, CD22+: CD22-positive, CD30+: CD30-positive, cHL: classical Hodgkin lymphoma, CPX-351: liposomal formulation of daunorubicin and cytarabine, DA: daunorubicin, Ara-C, DA-EPOCH: etoposide, doxorubicin, vincristine, prednisone, cyclophosphamide, DLTs: dose-limiting toxicities, EFS: event-free survival, FLAG-Ida: fludarabine, cytarabine, InO: inotuzumab ozogamicin, MIRV: mirvetuximab soravtansine, MTD: maximum tolerated dose, ORR: objective response rate, OS: overall survival, Ph-: Philadelphia chromosome-negative, R/R: relapsed or refractory, RP2D: recommended phase II dose, TNBC: triple-negative breast cancer, TV: tisotumab vedotin.

#### 2.1.2. ADCs Combined with Immune Checkpoint Inhibitors (ICIs)

While ICIs have shown substantial progress in cancer treatment, many patients still do not benefit from ICI monotherapy. To overcome resistance and enhance clinical efficacy, combination strategies with ADCs are being actively explored. ADCs can induce immunogenic cell death, activate T cells, and reshape the tumor microenvironment, creating conditions favorable for immune response [42,53,54,55]. ICIs such as anti-PD-1 (pembrolizumab, nivolumab, avelumab), anti-PD-L1 (durvalumab, atezolizumab), and anti-CTLA-4 (ipilimumab) can further amplify this response by releasing immune suppression [53]. As a result, ADC–ICI combinations are being widely investigated as a promising therapeutic strategy across various cancers. Table 3 summarizes the clinical trials on ADC-ICIs combination regimens.

***Brentuximab vedotin***

The combination of BV with ICIs has been actively investigated in NSCLC, melanoma, and across a range of malignancies, particularly in CD30-positive lymphomas.

In the SGN35-033 phase II trial, BV was combined with pembrolizumab, the PD-1 inhibitor, among patients with metastatic NSCLC (mNSCLC) or metastatic melanoma previously treated with PD-1 inhibitors. The ORR was 14% in secondary-resistant NSCLC and 24% in secondary-resistant melanoma, with responses being durable and clinically meaningful. Further evaluation in first-line NSCLC and head and neck squamous cell carcinoma is ongoing [56].

BV has also been studied combined with the PD-1 inhibitor nivolumab in several trials, including the ACCRU RU0515051 phase II trial in older or chemotherapy-ineligible cHL patients [57], the phase II trial (BV+nivolumab post-HSCT) in high-risk R/R cHL [58], the CheckMate 436 phase I/II trial in primary mediastinal large B-cell lymphoma (PMBL) [59], and the SGN35-015 phase II trial in older patients with advanced cHL [44]. Notably, the CheckMate 744 phase II study in children, adolescents, and young adults (CAYA) with R/R cHL showed that BV plus nivolumab induction achieved a complete metabolic response (CMR) rate of 59% without conventional chemotherapy, underscoring its potential as an effective and well-tolerated first salvage regimen in this population [60].

In the ECOG-ACRIN E4412 phase II trial in R/R cHL, BV plus nivolumab (BV/N) and BV plus nivolumab with the CTLA-4 inhibitor ipilimumab (BV/N/I) were compared. The complete response (CR) rates were 60.7% and 66.7%, respectively. At a median follow-up of 24 months, mPFS and duration of response (DOR) were not reached, indicating that both regimens provided durable and favorable disease control [61].

These regimens also maintained acceptable tolerability, with immune-related events and neuropathy occurring at frequencies consistent with established profiles.

***Trastuzumab emtansine***

Trastuzumab emtansine (T-DM1) is an ADC that targets HER2-positive cancer cells to deliver the cytotoxic agent DM1, causing tumor cell death and antigen release. When combined with the PD-L1 inhibitor atezolizumab, this immune-activating effect can be amplified by restoring T-cell activity and overcoming tumor-induced immune suppression.

The ASTEFANIA phase III trial is enrolling high-risk HER2-positive early breast cancer (EBC) patients to evaluate the efficacy and safety of this combination. As of 28 June 2023, 789 of the planned 1700 patients had been enrolled. Participants are randomized to receive either T-DM1 plus atezolizumab or T-DM1 plus placebo, with invasive disease-free survival (iDFS) as the primary endpoint [62]. In addition, the MyTACTIC phase II trial evaluated targeted therapies in advanced solid tumors with actionable biomarkers; in Arm F, atezolizumab plus T-DM1 was assessed for confirmed objective response rate (cORR) and demonstrated antitumor activity [63].

Reported adverse events largely reflected the known T-DM1 toxicity spectrum, including thrombocytopenia and mild hepatic enzyme elevations, which were generally manageable with routine monitoring.

***Sacituzumab govitecan***

Sacituzumab govitecan (SG) is a Trop-2-targeting ADC that has been actively investigated for its clinical efficacy and safety in combination with various ICIs. Ongoing studies are evaluating SG with agents such as pembrolizumab, avelumab, nivolumab, and atezolizumab across multiple cancer types, with the potential to offer new therapeutic strategies that improve patient outcomes.

The TROPHY-U-01 phase II trial in metastatic urothelial carcinoma (mUC) after platinum-based chemotherapy tested SG plus pembrolizumab, achieving an ORR of 41%, which was higher than the ~21% reported with pembrolizumab monotherapy [64]. In treatment-naïve mNSCLC with high PD-L1 expression (≥50%), the EVOKE-02 phase II trial demonstrated promising activity, with an ORR of 67% and manageable adverse events; based on these results, a larger confirmatory phase III study (EVOKE-03) is currently ongoing [65]. In residual triple-negative breast cancer (TNBC), the OptimICE-RD phase III trial is underway, with iDFS as the primary endpoint [66]. In previously untreated PD-L1-positive (CPS ≥ 10) locally advanced unresectable or metastatic TNBC, the ASCENT-04/KEYNOTE-D19 phase III trial showed that SG plus pembrolizumab achieved a mPFS of 11.2 months compared with 7.8 months for chemotherapy plus pembrolizumab, representing a statistically and clinically meaningful improvement and supporting the combination as a potential new standard of care in this setting [67].

The JAVELIN Bladder Medley phase II trial in the maintenance setting for locally advanced or metastatic urothelial carcinoma (la/mUC) patients who had no disease progression after receiving first-line platinum-based chemotherapy assigned 74 of 111 enrolled patients to the avelumab plus SG arm, demonstrating a significant PFS benefit (median 11.17 vs. 3.75 months with avelumab monotherapy) without new safety signals [68].

According to Mar et al. (2025), another ongoing phase II trial is evaluating adjuvant SG in combination with nivolumab in muscle-invasive urothelial carcinoma at high risk of recurrence, with 6-month disease-free survival (DFS) as the primary endpoint and a target enrollment of 23 patients [69].

Furthermore, as reported by Jain et al. (2024), a phase I/II study in metastatic cisplatin-ineligible mUC evaluated SG combined with ipilimumab and nivolumab, where the RP2D determined in phase I achieved an impressive ORR of 88.2% [70].

The MORPHEUS-panBC trial is an ongoing phase Ib/II study evaluating various treatment combinations in patients with locally advanced or metastatic TNBC. In an interim analysis, the primary endpoints were ORR and safety, with an ORR of 76.7%. Although PFS data are still immature, the atezolizumab plus SG arm showed an advantage (12.2 vs. 5.9 months), and the safety profile was consistent with individual agents without new signals [71]. Similarly, the SMART phase II trial is currently ongoing, assessing the efficacy and safety of SG, either as monotherapy or in combination with atezolizumab, in patients with rare genitourinary (GU) tumors. The primary endpoint is the ORR for each cohort [72].

The safety findings were consistent with SG’s established profile, with hematologic and gastrointestinal toxicities observed but typically manageable with supportive care.

***Enfortumab vedotin***

Enfortumab vedotin (EV) is an ADC targeting nectin-4, and it has already been approved as a monotherapy for previously treated patients with advanced urothelial carcinoma, as well as in combination with an ICI for treatment-naïve patients. Based on its therapeutic potential, EV is also being actively investigated in rare GU tumors.

The EV-103 cohort K study was a phase I/II clinical trial evaluating the initial efficacy and safety of EV in combination with pembrolizumab in patients with previously untreated la/mUC who were ineligible for cisplatin-based chemotherapy. Among 149 patients enrolled, 76 patients received the combination therapy. The cORR was 64.5%, with a mDOR not reached and 65.4% of responders maintaining their response at 12 months. Adverse events were generally manageable, and no new safety signals were observed. These findings demonstrated the potential of the combination therapy and laid the foundation for the subsequent EV-302 trial [73]. The EV-302 study was a randomized phase III clinical trial conducted in a similar patient population, directly comparing the efficacy and safety of EV in combination with pembrolizumab to standard chemotherapy. A total of 886 patients were enrolled, with 442 patients assigned to the combination therapy group. Key results showed significantly improved outcomes with the combination therapy, with mPFS (12.5 months vs. 6.3 months) and median OS (31.5 months vs. 16.1 months) both favoring the combination over chemotherapy. The incidence of grade ≥ 3 treatment-related adverse events was lower in the combination group compared to the chemotherapy group. These significant results confirmed the promising findings of EV-103 and led to the FDA approval of the combination therapy as a first-line treatment for la/mUC on 15 December 2023, establishing it as a new standard of care [74]. Building on this success, the E-VIRTUE phase II clinical trial was planned to evaluate the combination of EV and pembrolizumab in patients with rare GU tumors. These include locally advanced or metastatic urothelial adenocarcinoma, urothelial squamous cell carcinoma, and refractory germ cell tumors (GCTs), which are tumors with limited treatment options. The trial is designed based on the hypothesis that EV-based therapies will demonstrate clinical activity in these rare tumor types. The primary endpoint is ORR, and final results have not yet been reported [75,76].

These regimens also showed predictable and manageable toxicities, with dermatologic reactions and metabolic disturbances remaining the most common adverse events.

***Trastuzumab deruxtecan***

Trastuzumab deruxtecan (T-DXd) is a HER2-targeted ADC. When combined with ICIs such as durvalumab, nivolumab, and pembrolizumab, it is expected to enhance tumor cell killing and tumor immunogenicity through synergistic effects, and multiple clinical trials are currently underway across different tumor types.

The BEGONIA study is an ongoing trial evaluating durvalumab in combination with novel agents in patients with HR-negative, HER2-low advanced/metastatic breast cancer (a/mBC). Schmid et al. (2023) reported outcomes in patients treated with T-DXd plus durvalumab, 46 patients were included in the efficacy analysis. The cORR was 57%, with responses observed regardless of PD-L1 expression. The mPFS was 12.6 months, with the upper bound of the confidence interval not reached, suggesting durable clinical benefit. These findings indicate that T-DXd plus durvalumab may significantly delay disease progression in HR-negative, HER2-low metastatic breast cancer (mBC), providing encouraging evidence for this combination [77]. The HUDSON study evaluated the efficacy and safety of T-DXd plus durvalumab in NSCLC patients with HER2 overexpression (HER2e) or HER2 mutations (HER2m) whose disease had progressed on prior ICI therapy. A total of 43 patients were enrolled (HER2e, *n* = 23; HER2m, *n* = 20). The ORR was 26.1% in HER2e and 35% in HER2m, supporting the potential activity of this combination in NSCLC [78]. In parallel, the TRUDI study is an ongoing phase II neoadjuvant trial in inflammatory breast cancer (IBC). Patients are stratified into two cohorts: Cohort 1, HER2-positive disease (HER2 IHC 3+ or 2+/ISH amplified), and Cohort 2, HER2-low disease (HER2 IHC 1+ or 2+/ISH non-amplified). The primary endpoint is pathologic complete response (pCR). Notably, TRUDI represents the first and only ongoing trial combining an anti-HER2 ADC with immunotherapy in HER2-expressing IBC, highlighting a novel therapeutic approach [79].

DS8201-A-U105 is a phase Ib study evaluating the safety and efficacy of T-DXd plus nivolumab in patients with HER2-expressing mBC and mUC. In Part 1, the recommended dose for expansion (RDE) of T-DXd 5.4 mg/kg plus nivolumab 360 mg was established, and in Part 2, efficacy was assessed at this dose, showing an ORR of 65.6% in Cohort 1 (HER2+ mBC), 50.0% in Cohort 2 (HER2-low mBC), and 36.7% in Cohort 3 (HER2-high mUC), while Cohort 4 (HER2-low mUC) was not evaluable due to small sample size. The combination demonstrated promising antitumor activity with no new safety concerns identified [80].

DS8201-A-U106 is a phase Ib study evaluating T-DXd plus pembrolizumab in mBC and NSCLC. The primary endpoint was confirmed ORR, with a total of 56 mBC patients (Cohorts 1 and 2) and 55 NSCLC patients (Cohorts 3 and 4) included. As of the data cutoff (18 November 2023), the cORR was 80.0% in Cohort 1 (HER2+ mBC), 23.1% in Cohort 2 (HER2-low mBC), 54.5% in Cohort 3 (HER2e NSCLC), and 66.7% in Cohort 4 (HER2m NSCLC). These results demonstrate that T-DXd plus pembrolizumab exerts meaningful antitumor activity in both mBC and NSCLC [81,82].

As in prior monotherapy studies, interstitial lung disease remained an important safety consideration, requiring vigilant observation during treatment.

***Tisotumab vedotin***

TV, an ADC targeting TF, is expected to provide enhanced antitumor activity not only in combination with chemotherapy but also with ICIs, and several studies are underway to evaluate this strategy.

The innovaTV 205/GOG-3024/ENGOT-cx8 trial is a phase Ib/II study conducted in patients with r/mCC, evaluating the safety and antitumor activity of TV in combination with pembrolizumab and other therapies. In the dose-escalation phase, the RP2D was determined as TV 2 mg/kg plus pembrolizumab 200 mg administered intravenously on day 1 of every 3-week cycle. In the dose-expansion phase, the ORR was 40.6% among 32 patients treated in the first-line setting and 35.3% among 34 patients treated in the second- or third-line setting. These findings indicate that the combination of TV and pembrolizumab showed an acceptable safety profile and promising antitumor activity in patients with r/mCC [42].

***Mirvetuximab soravtansine***

MIRV targets FRα, a biomarker overexpressed in up to 95% of ovarian cancers. MIRV has demonstrated antitumor activity and tolerability across various combination regimens in patients with recurrent ovarian cancer, and is continuously being investigated as a potential combination therapy.

In the phase Ib/II trial by Matulonis et al. (2025), the safety and efficacy of MIRV in combination with pembrolizumab were evaluated in patients with platinum-resistant ovarian cancer (PROC). Among 55 patients, the ORR was 31%, with a mDOR of 8.0 months. Notably, higher levels of FRα expression were associated with longer mDOR [83]. In addition, a phase II clinical trial by Porter et al. (2024) evaluated the combination of MIRV and pembrolizumab in 16 patients with recurrent or persistent, MSS/pMMR, FRα-positive endometrial cancer (EC). The primary endpoint ORR was 37.5%, and 12.5% of patients achieved 6-month PFS. The study met its co-primary endpoints, supporting the combination as worthy of further investigation [84].

***Datopotamab deruxtecan***

Datopotamab deruxtecan (Dato-DXd), a TROP2-targeting ADC, is currently being actively investigated in breast cancer and NSCLC, particularly in combination therapy settings. The combination therapy of Dato-DXd and the ICIs has shown meaningful results across various clinical trials targeting TNBC.

The BEGONIA Phase Ib/II study evaluated the safety and efficacy of Dato-DXd in combination with durvalumab in 62 previously untreated patients with advanced or metastatic TNBC. In arm 7, which included patients with PD-L1 low-expressing tumors, the ORR reached 79%, highlighting the potential of the combination therapy. The safety profile was consistent with previous findings, and arm 8, which includes patients with PD-L1 positive tumors, is currently ongoing [85]. In parallel, the I-SPY2.2 phase II trial enrolled 106 high-risk stage II/III breast cancer patients to assess the efficacy of Dato-DXd in combination with durvalumab. The combination therapy achieved a pCR rate of 54%, without any newly observed toxicities. These results support the potential of the regimen in early-stage breast cancer patients [86]. Encouraged by the positive outcomes of BEGONIA and I-SPY2.2, several phase III clinical trials have been launched. TROPION-Breast03 is currently evaluating patients with stage I–III TNBC who have residual invasive disease after neoadjuvant therapy. The study compares Dato-DXd monotherapy or its combination with durvalumab against standard treatment, with a total of 1075 patients randomized. The primary endpoint is iDFS [87]. Additionally, TROPION-Breast04 is a phase III clinical trial examining the efficacy and safety of neoadjuvant Dato-DXd and durvalumab, followed by adjuvant durvalumab treatment in patients with early-stage TNBC or HR-low/HER2-negative breast cancer. A total of 1728 patients will be randomized. The primary endpoints are pCR and EFS [88]. Lastly, TROPION-Breast05 targets patients with PD-L1 high-expressing, unresectable locally recurrent or metastatic TNBC. This phase III trial compares the efficacy and safety of Dato-DXd plus durvalumab versus the standard of care, which consists of chemotherapy in combination with pembrolizumab. A total of 625 patients will be randomized, and the primary endpoint is PFS [89].

Beyond breast cancer, Dato-DXd is also being actively investigated in NSCLC, particularly in combination with ICIs.

TROPION-Lung08 is a phase III clinical trial evaluating the safety and efficacy of Dato-DXd in combination with pembrolizumab compared to pembrolizumab monotherapy as a first-line treatment in patients with a/mNSCLC without actionable genomic alterations (AGAs). The study plans to enroll 740 patients, with PFS as assessed by BICR and OS as the primary endpoints. The trial is currently ongoing [54].

Separately, Cohort 5 of the TROPION-Lung04 study investigated the safety and preliminary efficacy of Dato-DXd in combination with rilvegostomig, a PD-1/CTLA-4 bispecific antibody, in patients with a/mNSCLC without AGAs. As of 24 October 2024, all 40 patients experienced treatment-emergent adverse events (TEAEs), and the ORR was reported as 57.5%. This combination demonstrated promising antitumor activity as a potential first-line option in this patient population [90]. The same drug combination is being further evaluated in the TROPION-Lung10 phase III study, which targets patients with non-squamous a/mNSCLC who are PD-L1 high expressors (tumor cell expression ≥ 50%) and do not have AGAs. The study aims to enroll 675 patients, with PFS as assessed by BICR and OS serving as the primary endpoints. Patient enrollment is currently in progress [91].

Across these studies, Dato-DXd-based combinations have shown manageable toxicity profiles, with stomatitis and hematologic adverse events occurring at expected frequencies and generally controllable with routine supportive measures.

**Table 3:** Summary of clinical trials on ADC-ICIs combination regimens. This table provides an overview of partner drugs, indications, primary efficacy and safety outcomes, clinical trial details, and publication year.

ADC	Target	Partner Drug	Indication	Primary Endpoint		Phase	NCT Number	Publication Year	Ref.
				Efficacy	Safety				
Brentuximab Vedotin	CD30	Ipilimumab, Nivolumab	R/R cHL	CR rate BV/N/I 66.7% BV/N 60.7%		Phase I Phase II	NCT01896999	2023	[61]
		Nivolumab	After autologous SCT in patients with high-risk cHL	18 months PFS 94%		Phase II	NCT03057795	2023	[58]
			R/R HL	CMR 59%		Phase II	NCT02927769	2023	[60]
			R/R PMBL	ORR 73.3%		Phase I Phase II	NCT02581631	2023	[59]
			Avanced-stage cHL in patients aged ≥60 years who are unfit for conventional chemotherapy	ORR 86%		Phase II	NCT01716806	2024	[44]
			Older patients with untreated HL	ORR 61%		Phase II	NCT02758717	2025	[57]
		Pembrolizumab	PD-1–pretreated mNSCLC and metastatic cutaneous melanoma	ORR secondary refractory mNSCLC 14% secondary refractory metastatic cutaneous melanoma 24%		Phase II	NCT04609566	2025	[56]
Trastuzumab Emtansine	HER2	Atezolizumab	Advanced solid tumors with HER2 amplification or mutation plus TMB-H/MSI-H/dMMR	cORR 12%		Phase II	NCT04632992	2024	[63]
			mUC	iDFS: Immature		Phase III	NCT04873362	2024	[62]
Sacituzumab Govitecan	Trop-2	Atezolizumab	Rare GU tumors such as small-cell, adenocarcinoma, and squamous cell bladder/urinary tract cancer, RMC and penile cancer	ORR: Immature		Phase II	NCT06161532	2024	[72]
			PD-L1+, inoperable locally a/mTNBC	ORR 76.7%		Phase Ib Phase II	NCT03424005	2024	[71]
		Avelumab	Locally a/m UC	mPFS 11.17 months		Phase II	NCT05327530	2025	[68]
		Ipilimumab, Nivolumab	Metastatic cisplatin-ineligible UC	ORR 88.2%	RP2D SG 8 mg/kg, Ipilimumab 3 mg/kg, Nivolumab 1 mg/kg	Phase I Phase II	NCT04863885	2024	[70]
		Nivolumab	Muscle-invasive UC at high-risk recurrence	6-month DFS: Immature		Phase II	NCT06682728	2025	[69]
		Pembrolizumab	mUC	ORR 41%		Phase II	NCT03547973	2024	[64]
			HR+/HER2- mBC	mPFS 8.4 months		Phase II	NCT04448886	2024	[92]
			mNSCLC with PD-L1 ≥ 50%	ORR 67%		Phase II	NCT05186974	2024	[65]
			TNBC with residual invasive disease after neoadjuvant therapy and surgery	iDFS: Immature		Phase III	NCT05633654	2024	[66]
			Previously untreated PD-L1-positive locally advanced inoperable or mTNBC	mPFS 11.2 months		Phase III	NCT05382286	2025	[67]
Enfortumab Vedotin	Nectin-4	Pembrolizumab	Locally a/m UC	cORR 64.5%		Phase I Phase II	NCT03288545	2023	[73]
			Locally a/m UC	mPFS 12.5 months, mOS 31.5 months		Phase III	NCT04223856	2024	[74]
			Rare GU tumors	ORR: Immature		Phase II	NCT06041503	2024	[75]
Trastuzumab Deruxtecan	HER2	Durvalumab	mNSCLC	ORR 26.1%		Phase II	NCT03334617	2023	[78]
			Unresectable locally HR-, HER2-low a/m BC		Grade 3/4 AEs 32%; Grade 5: 1 case	Phase I Phase II	NCT03742102	2023	[77]
			Stage III HER2-expressing Inflammatory BC	pCR: Immature		Phase II	NCT05795101	2024	[79]
		Nivolumab	HER2-expressing mBC or mUC	cORR Part 2 mBC cohort 1 65.6%, cohort 2 50.0% mUC cohort 3 cORR 36.7%, cohort 4 cORR: Immature	Part 1 MTD 5.4 mg/kg	Phase I	NCT03523572	2024	[80]
		Pembrolizumab	IO-naive HER2-expressing or HER2 mutant NSCLC	cORR cohort 3(HER2E) 54.5% cohort 4(HER2m) 66.7%		Phase Ib	NCT04042701	2024	[81]
			HER2+ or HER2-low a/m BC	cORR cohort 1(HER2+) 80.0% cohort 2(HER2-low) 23.1%		Phase Ib	NCT04042701	2025	[82]
Tisotumab Vedotin	TF	Pembrolizumab	Recurrent or metastatic Cervical Cancer	ORR 1 L treatment 40.6% 2 L, 3 L treatment 35.3%	DLTs 0%, RP2D TV 2 mg/kg on Day 1, Pembrolizumab 200 mg on Day 1	Phase I Phase II	NCT03786081	2023	[42]
Mirvetuximab Soravtansine	FRα	Pembrolizumab	MSS recurrent or persistent endometrial cancer	ORR 37.5%, 6-month PFS 12.5%		Phase II	NCT03835819	2024	[84]
			platinum-resistant ovarian cancer	ORR 31%		Phase I Phase II	NCT02606305	2025	[83]
Datopotamab Deruxtecan	Trop-2	Durvalumab	Arm 7: a/m TNBC Arm 8: PD-L1 positive a/m TNBC	ORR Arm 7 Part 2 74% Arm 8 Part 2 -	Grade ≥ 3 TRAEs Arm 7 Part 1 57% Arm 8 Part 1 -	Phase I Phase II	NCT03742102	2023	[85]
			TNBC and residual invasive disease at surgical resection after neoadjuvant therapy	iDFS: Immature		Phase III	NCT05629585	2024	[87]
			EBC	pCR 54%		Phase II	NCT01042379	2024	[86]
			Early-stage TNBC or HR-low/HER2-negative BC	pCR, EFS: Immature		Phase III	NCT06112379	2025	[88]
			PD-L1-high locally recurrent inoperable or mTNBC	PFS: Immature		Phase III	NCT06103864	2025	[89]
		Pembrolizumab	a/mNSCLC	PFS, OS: Immature		Phase III	NCT05215340	2023	[54]
		Rilvegostomig	Locally a/m nonsquamous NSCLC	PFS, OS: Immature		Phase III	NCT06357533	2025	[91]
			a/mNSCLC		Grade ≥ 3 TRAEs 60%	Phase Ib	NCT04612751	2025	[90]

a/m: advanced/metastatic, AEs: adverse events, BC: breast cancer, BV/N: brentuximab vedotin + nivolumab, BV/N/I: brentuximab vedotin, nivolumab, ipilimumab, cHL: classical Hodgkin lymphoma, CMR: complete molecular response, cORR: confirmed objective response rate, CR: complete response, DFS: disease-free survival, DLTs: dose-limiting toxicities, EBC: early stage breast cancer, GU: genitourinary, HER2E: HER2 overexpression, HER2m: HER2 mutations, HL: Hodgkin lymphoma, iDFS: invasive disease-free survival, Immature: data not yet mature for analysis, IO: immuno-oncology, mBC: metastatic breast cancer, mNSCLC: metastatic non-small-cell lung cancer, mOS: median overall survival, mPFS: median progression free survival, MSS: microsatellite stable, MTD: maximum tolerated dose, mTNBC: metastatic triple-negative breast cancer, mUC: metastatic urothelial carcinoma, NSCLC: non-small-cell lung cancer, ORR: objective response rate, pCR: pathologic complete response, PFS: progression free survival, PMBL: primary mediastinal large B-cell lymphoma, RMC: renal medullary carcinoma, R/R: relapsed or refractory, RP2D: recommended phase II dose, SCT: stem cell transplantation, SG: sacituzumab govitecan, TNBC: triple-negative breast cancer, TRAEs: treatment-related adverse events, TV: tisotumab vedotin, UC: urothelial carcinoma.

#### 2.1.3. ADCs Combined with Molecular Targeted Cancer Therapies

In recent years, ADCs have been actively investigated in combination with various molecular targeted therapies to enhance antitumor efficacy. This approach is driven by the clinical need for more effective treatment options in advanced or R/R cancers, where current therapies often show limited durability or suboptimal outcomes. Molecular targeted agents offer precision in targeting tumors based on specific genetic alterations or biomarkers, and when combined with ADCs, they can provide complementary mechanisms that improve therapeutic outcomes [93,94]. These combinations may help overcome resistance mechanisms such as reduced antigen expression or signaling bypass, and offer safer, more effective strategies for older or vulnerable patients [95,96]. As a result, clinical research on ADC-based combination therapies is rapidly expanding across multiple tumor types. Table 4 summarizes the clinical trials on ADC-molecular targeted cancer therapy combination regimens.

***Brentuximab vedotin***

Recent studies have shown that BV, a CD30-targeting ADC, is being actively explored in combination with other molecularly targeted agents to enhance therapeutic efficacy.

In a phase II trial reported by Mei et al. (2024), 36 patients with R/R Hodgkin lymphoma (HL) were treated with a combination of BV and ibrutinib. The study demonstrated a CR rate of 33% [97].

The ECHELON-3 study, a phase III randomized trial, evaluated the efficacy and safety of BV plus lenalidomide (Len) and rituximab (R) in those with R/R diffuse large B-cell lymphoma (DLBCL). Among 230 randomized patients, 112 were included in the efficacy analysis. The combination therapy significantly improved outcomes compared to the control group (placebo + Len + R). The median OS was 13.8 months in the BV + Len + R arm versus 8.5 months in the control arm, corresponding to a 37% reduction in the risk of death. Moreover, both the ORR (64% vs. 42%) and the CR rate (40% vs. 19%) favored the experimental arm. These findings suggest that the BV + Len + R regimen may represent a potential treatment option for patients who are ineligible for, or relapse after, stem cell transplantation (SCT), CAR T-cell therapy, or bispecific antibody therapy. Reflecting this clinical benefit, the regimen received FDA approval on 11 February 2025, for the treatment of those with R/R large B-cell lymphoma (LBCL) [98].

***Trastuzumab emtansine***

T-DM1, a HER2-targeting ADC, has been actively investigated in combination with various molecularly targeted therapies. For example, the TRAEMOS trial explored the combination of T-DM1 with osimertinib [99], and other ongoing clinical studies are evaluating combinations with agents such as neratinib and tucatinib.

In the phase III HER2CLIMB-02 study, tucatinib in combination with T-DM1 was assessed for efficacy and safety in patients with pretreated HER2-positive locally advanced or metastatic breast cancer (HER2+ la/mBC). As of the data cutoff on 29 June 2023, the tucatinib arm (*n* = 228) demonstrated a mPFS of 9.5 months, representing a statistically significant improvement. Notably, in patients with brain metastases, the hazard ratio (HR) for disease progression was 0.639, indicating a meaningful reduction in the risk of progression and reinforcing the therapeutic potential of this regimen for HER2+ breast cancer patients with brain metastases [100]. In addition, the TUCATEMEB phase II trial is ongoing, evaluating the safety and efficacy of the same combination (tucatinib + T-DM1) in patients with HER2-positive solid tumors and active brain metastases. The primary endpoint is the assessment of intracranial antitumor activity according to modified RECIST criteria. Patient enrollment is ongoing, and no results have yet been reported [101].

The TBCRC 022 phase II trial (cohort 4) assessed the efficacy and safety of neratinib plus T-DM1 in those with HER2-positive breast cancer brain metastases (BCBM). The study included three subgroups: cohort 4A (no prior CNS-directed therapy), cohort 4B (prior CNS-directed therapy but no prior T-DM1), and cohort 4C (both prior CNS-directed therapy and T-DM1), with intracranial ORR by Response Assessment in Neuro-Oncology-Brain Metastases (RANO-BM) of 33.3%, 35.3%, and 28.6%, respectively. These findings indicate that the neratinib + T-DM1 combination exhibits intracranial antitumor activity, suggesting a potential synergistic effect of neratinib with T-DM1 [102]. For reference, the NSABP FB-10 phase Ib/II trial also evaluated T-DM1-based combinations in patients with metastatic HER2-positive breast cancer, reporting an ORR of 32% in the phase II portion. Collectively, these data underscore that T-DM1-based combination strategies are being actively explored across different HER2-positive settings, including patients with brain metastases [103].

These regimens were generally well tolerated, with thrombocytopenia and hepatic laboratory abnormalities occurring as anticipated.

***Inotuzumab ozogamicin***

InO, a CD22-targeting ADC, has been explored in combination with various targeted therapies to develop tailored strategies for acute lymphoblastic leukemia (ALL) according to patient age and disease status.

A phase I trial evaluated the combination of venetoclax and InO in adult patients with R/R ALL. All nine patients achieved a complete remission, and venetoclax at 400 mg administered for 21 days in combination with standard-dose InO was well tolerated, establishing the RP2D without major safety concerns [104].

In addition, the Alliance A041703 phase II trial investigated the combination of InO and blinatumomab in elderly patients with newly diagnosed, CD22-positive B-cell ALL. The majority were Ph-negative, while two patients had Ph-positive disease. For the entire cohort (*n* = 33), the study met its predefined success criterion, reporting a 1-year EFS rate of 75%, thereby highlighting the potential of this regimen as an effective treatment strategy in this high-risk population [105].

While efficacy was notable, the known hepatotoxicity profile of InO necessitated careful monitoring, particularly regarding VOD risk.

***Polatuzumab vedotin***

Polatuzumab vedotin (Pola) has emerged as a promising therapeutic partner in the treatment of B-cell lymphomas, including DLBCL.

In Chavez et al. (2024), subcutaneous (SC) mosunetuzumab combined with Pola showed promising efficacy in 40 patients with R/R DLBCL, with an ORR of 78% and a CR rate of 58% [106]. Based on these findings, Olszewski et al. (2023) evaluated the intravenous (IV) formulation in previously untreated elderly and unfit/frail patients, showing a BOR of 80% and an end-of-treatment ORR of 55%. Safety was confirmed by an Independent Review Committee (IRC), supporting the potential of M-Pola in this population [107]. Building upon these findings, the phase III SUNMO study was conducted to directly compare the combination of mosunetuzumab SC and polatuzumab vedotin (M-Pola) with the standard Rituximab, Gemcitabine and Oxaliplatin (R-GemOx) regimen in patients with R/R DLBCL. As of 17 February 2025, among 208 randomized patients, the mPFS was 11.5 months in the M-Pola group (*n* = 138), showing a marked improvement compared to 3.8 months in the R-GemOx group (*n* = 70). The ORR was also significantly higher in the M-Pola group (70% vs. 40%), indicating that the M-Pola combination may serve as a new standard of care for R/R DLBCL [108]. In addition, Study 3, a phase Ib/II trial involving 120 patients with aggressive R/R LBCL, established the RP2D for the M-Pola regimen, and the expansion cohort reported an IRC-assessed ORR of 59.2%, confirming consistent efficacy [109].

Pola has also been evaluated in combination with venetoclax and either obinutuzumab or rituximab in patients with R/R follicular lymphoma (FL) and DLBCL. In this phase Ib/II study, the CR rate at the end of induction was 59.2% in the FL cohort (*n* = 74) and 31.3% in the DLBCL cohort (*n* = 57). These results demonstrated promising activity and acceptable safety, highlighting the potential for broader clinical applications of Pola-based regimens [110].

In a separate phase II study involving 24 treatment-naïve elderly and unfit/frail DLBCL patients, the combination of Pola, zanubrutinib, and rituximab (Pola-ZR) was evaluated. Following six cycles of therapy, both ORR and CR rates were reported at 83%, reflecting deep and meaningful responses [111].

The Pola-R-Len regimen was studied in the phase Ib/II GO29834 trial in transplant-ineligible R/R DLBCL patients. In the phase Ib portion (*n* = 18), the RP2D of lenalidomide was established as 20 mg. Among 39 patients enrolled in the phase II portion, the IRC-assessed CR rate was 31% [112]. In an independent phase II study conducted in frail or elderly DLBCL patients with limited mobility, the same regimen was administered. As of 20 July 2024, a total of 21 patients had been enrolled, and after a median follow-up of 6 months, 8 patients had completed treatment, with a 100% CR rate at the EOT [113].

Additionally, a phase Ib/II trial of glofitamab plus Pola in patients with R/R LBCL enrolled 129 patients, with efficacy assessed in 128. The best ORR was 80% and the CR rate was 62%. This combination demonstrated sustained responses and an overall manageable safety profile [114].

These findings highlight the broad utility of Pola in combination therapies, from frontline use in unfit elderly patients to salvage treatment in refractory B-cell lymphoma. These regimens were generally well tolerated, with cytopenias and peripheral neuropathy representing the most common but manageable adverse events.

***Enfortumab vedotin***

EV, an ADC targeting nectin-4, continues to be investigated in combination strategies for mUC, a disease that remains highly lethal despite recent therapeutic advances.

The ETCTN 10483 phase I trial evaluated EV in combination with erdafitinib (E) in patients with FGFR3/2 genomic alterations (GAs) whose disease had progressed after platinum-based chemotherapy and/or PD-1/PD-L1 inhibitors. Among nine patients assessed for dose-limiting toxicities (DLTs), the RP2D of EV was determined to be 1.25 mg/kg, and all nine patients achieved an ORR, confirming antitumor activity [115].

In addition, a phase I/Ib trial investigated the combination of EV and cabozantinib. As of 6 January 2024, ten patients had been treated, with nine evaluable patients demonstrating an ORR of 88.9%. Grade ≥ 3 treatment-related adverse events were observed but were consistent with the expected safety profiles of each agent. Taken together, both studies demonstrated promising early antitumor activity with manageable safety profiles, supporting the potential of EV-based combination strategies in the treatment of mUC [116].

***Trastuzumab deruxtecan***

Currently, a wide range of early-phase clinical trials are exploring T-DXd in combination with molecularly targeted agents and other therapies across different tumor types.

DESTINY-Breast07 is an ongoing phase Ib/II trial evaluating the safety, tolerability, and efficacy of T-DXd combined with pertuzumab in those with previously untreated HER2-positive mBC. According to data as of 22 December 2023, the combination demonstrated a cORR of 84% and a 12-month PFS rate of 89.4%, showing robust antitumor activity with no unexpected safety concerns [117,118].

Another early-phase study, the DS3201-324 phase Ib trial, is evaluating the combination of valemetostat and T-DXd in patients with HER2-low breast cancer, gastric cancer (GC)/gastroesophageal junction (GEJ) adenocarcinoma, and NSCLC. The study aims to assess enhanced efficacy while maintaining safety, with approximately 70 patients per subprotocol planned for enrollment. Patient recruitment is currently ongoing [119,120].

In addition, the Beamion BCGC-1 phase Ib/II trial is investigating zongertinib plus T-DXd in patients with HER2-positive metastatic gastroesophageal adenocarcinoma (mGEAC) or mBC. A total of 240 patients are planned for enrollment, with DLTs during the maximum tolerated dose evaluation period and ORR designated as the primary endpoints. Enrollment is ongoing [121].

***Sacituzumab govitecan***

Conventional cancer therapies are often limited by high toxicity. To overcome this, combination strategies using Trop-2-targeting SG and molecular targeted agents have gained attention for their potential to reduce toxicity while improving efficacy.

Abel et al. (2023) evaluated the safety and clinical feasibility of combining SG with the DNA damage response (DDR) inhibitor berzosertib in a phase I study enrolling patients with advanced solid malignancies. Twelve patients were enrolled, and no DLTs were observed, allowing dose escalation up to SG 10 mg/kg and berzosertib 210 mg/m^2^. Compared to traditional chemotherapy-DDR inhibitor combinations, this regimen demonstrated an improved safety profile, suggesting its potential as a novel therapeutic strategy [122].

McGregor et al. (2024) assessed the safety and efficacy of combining SG with EV, another standard ADC, in patients with mUC. Among 24 enrolled patients, 23 were analyzed. Based on results from three dose levels, the RP2D was determined to be SG 8 mg/kg and EV 1.25 mg/kg. The ORR was 70%. This study established a safe and active combination regimen, supporting the need for further investigation in subsequent trials [123].

***Loncastuximab tesirine***

Multiple clinical trials have evaluated the potential of loncastuximab tesirine (Lonca)-based combination therapies in those with R/R B-cell non-Hodgkin lymphoma (B-NHL).

First, the combination of Lonca and rituximab (Lonca-R) was assessed in a phase II study involving patients with R/R FL. As of September 2024, among 39 enrolled patients, the CR rate at week 12 was 67%, and the ORR was 97%, with no new safety concerns reported. These results suggest that Lonca-R may be a promising treatment option for R/R FL, and a multi-center expansion cohort is currently ongoing [124]. In addition, the phase III LOTIS-5 trial is evaluating Lonca-R in patients with R/R DLBCL. Preliminary data from the first 20 patients showed a mPFS of 8.3 months, an ORR of 80%, with CRs accounting for 50%, with a manageable safety profile. Patient enrollment is ongoing [125].

Meanwhile, LOTIS-7 is a phase Ib study evaluating Lonca in combination with other anticancer agents. Key treatment arms include combinations with polatuzumab vedotin (Arm C), glofitamab (Arm E), and mosunetuzumab (Arm F). As of April 2025, interim results from Arm E (Lonca + glofitamab) showed an ORR of 93.3% and a CR rate of 86.7% in 30 patients with R/R LBCL who had received at least two prior therapies. While these early results are promising, long-term outcomes are still under follow-up. Arm C has completed enrollment, but detailed outcomes have not yet been disclosed. Arm F and other arms are still actively enrolling patients [96].

The combination of Lonca with the CD3/CD20 bispecific antibody mosunetuzumab is also being investigated in a separate phase II trial for R/R DLBCL. A total of 26 patients are planned for enrollment. As of now, during the safety lead-in phase, three patients have been enrolled, and no DLTs have been observed. Primary endpoints include safety and ORR [95].

Additionally, a phase I/II multicenter, single-arm clinical trial is evaluating the combination of imvotamab and Lonca in patients with R/R NHL. In phase Ia, imvotamab monotherapy showed no DLTs. The ongoing phase II trial is assessing two dose levels (100 mg and 300 mg) in a randomized design. The phase Ib combination trial was planned to begin in 2023 and aims to explore the dual targeting of CD19 and CD20, potentially offering improved efficacy and safety over existing therapies [126].

The safety findings reflected expected cytopenias and liver function abnormalities typical of pyrrolobenzodiazepine-based ADCs.

***Tisotumab vedotin***

r/mCC remains a clinical challenge, with limited effective treatment options across both first- and second-line settings. To address this, the addition of molecularly targeted cancer therapies such as bevacizumab is being actively investigated to enhance treatment efficacy and expand therapeutic options.

The innovaTV 205/GOG-3024/ENGOT-cx8 study was a phase Ib/II clinical trial that evaluated the safety and antitumor activity of combining TV with bevacizumab in patients with r/mCC. In the dose-escalation cohort for bevacizumab, DLTs were assessed in 15 patients, and no DLTs were observed. As a result, the RP2D was determined to be 2 mg/kg for TV and 15 mg/kg for bevacizumab. The acceptable safety profile and encouraging antitumor activity observed in this study support the feasibility of adding TV to bevacizumab in this patient population [42].

***Mirvetuximab soravtansine***

MIRV, an FRα-targeting ADC, is being actively investigated in multiple clinical trials for its potential complementary activity when combined with molecularly targeted therapies with different mechanisms of action.

The phase Ib/II FORWARD II trial evaluated the antitumor activity and safety of MIRV in combination with bevacizumab in patients with PROC. The confirmed ORR in the overall population was 44%, with a mDOR of 9.7 months and a mPFS of 8.2 months. The safety profile was consistent with the known toxicities of each agent, indicating manageable tolerability. These encouraging findings supported the clinical potential of the MIRV–bevacizumab combination and ultimately served as the basis for designing the phase III GLORIOSA trial [127]. GLORIOSA is enrolling approximately 418 patients with FRα-high platinum-sensitive ovarian cancer (PSOC) to evaluate the potential benefit of MIRV plus bevacizumab as maintenance therapy compared with bevacizumab alone, with PFS per RECIST v1.1 as the primary endpoint [94].

***Datopotamab deruxtecan***

Dato-DXd is a TROP2-targeting ADC being investigated in a broad global clinical program across multiple tumor types, including NSCLC, TNBC, and HR-positive, HER2-negative breast cancer.

Among these, the DS3201-324 phase Ib trial is evaluating Dato-DXd in combination with valemetostat in patients with locally advanced, unresectable, or metastatic non-squamous NSCLC previously treated with at least two lines of therapy. The study includes safety/tolerability and ORR as the primary endpoints, and enrollment is ongoing [120].

The ORCHARD phase II study further explores Dato-DXd plus osimertinib in EGFR-mutated advanced NSCLC after progression on first-line osimertinib. Patients received Dato-DXd 4 or 6 mg/kg Q3W with osimertinib 80 mg QD, yielding ORRs of 43% and 36%, respectively, with improved DOR and mPFS favoring the 6 mg/kg cohort, thereby supporting it as the preferred starting dose in this setting [128]. In parallel, the phase III TROPION-Lung14 and TROPION-Lung15 trials are evaluating the same combination in broader contexts. TROPION-Lung14 randomizes ~562 untreated patients to osimertinib alone or with Dato-DXd in the first-line setting, while TROPION-Lung15 randomizes ~630 patients who progressed on osimertinib to Dato-DXd monotherapy, Dato-DXd plus osimertinib, or platinum-based chemotherapy. Both designate PFS by BICR as the primary endpoint. Collectively, these trials are expected to clarify the role of the Dato-DXd plus osimertinib combination across both frontline and post-progression settings in EGFR-mutated NSCLC. Disease progression after osimertinib is frequent, and combination strategies with broadly active agents such as ADCs may offer a means to overcome resistance [129,130].

***Telisotuzumab vedotin***

Teliso-V is an ADC targeting the c-Met protein and represents the only ADC to date selected based on a biomarker in EGFR-mutated NSCLC. Since c-Met overexpression occurs in approximately 50% of EGFR-mutated NSCLC tumors following osimertinib progression, targeting c-Met with an ADC was hypothesized to provide therapeutic benefit.

To evaluate this approach, a phase Ib study enrolled 38 patients with locally advanced or metastatic non-squamous NSCLC harboring EGFR mutations and c-Met overexpression who had progressed after prior osimertinib therapy, and assessed the combination of Teliso-V and osimertinib. At a median follow-up of 7.4 months, the independent central review (ICR)-assessed ORR was 50.0%, mPFS was 7.4 months, and median DOR was not reached (NR), demonstrating promising antitumor activity with a manageable safety profile. However, uncertainties remain regarding the optimal cutoff for defining c-Met overexpression, and limited efficacy was observed in patients with brain metastases. Consequently, the phase III trial was discontinued. Nonetheless, these findings highlight the feasibility of biomarker-driven ADC approaches in NSCLC and underscore their potential to inform rational treatment selection in the context of multiple available therapeutic options [93,131].

**Table 4:** Summary of clinical trials on ADC-molecular targeted cancer therapy combination regimens. This table provides an overview of partner drugs, indications, primary efficacy and safety outcomes, clinical trial details, and publication year.

ADC	Target	Partner Drug	Indication	Primary Endpoint		Phase	NCT Number	Publication Year	Ref.
				Efficacy	Safety				
Brentuximab Vedotin	CD30	Ibrutinib	R/R HL	CR rate 33%		Phase II	NCT02744612	2024	[97]
		Lenalidomide, Rituximab	R/R DLBCL	mOS 13.8 months		Phase III	NCT04404283	2025	[98]
Trastuzumab Emtansine	HER2	Neratinib	Pretreated and untreated HER2+ BC brain metastases	CNS ORR cohort 4A 33.3% cohort 4B 35.3% cohort 4C 28.6%		Phase II	NCT01494662	2024	[102]
			Women with metastatic HER2+ BC	ORR 32%		Phase Ib Phase II	NCT02236000	2024	[103]
		Osimertinib	EGFR mutation-positive NSCLC	ORR 4%		Phase II	NCT03784599	2023	[99]
		Tucatinib	a/m HER2+ BC	mPFS 9.5 months		Phase III	NCT03975647	2024	[100]
			HER2+ metastatic solid tumors and metastases to brain	Intracranial antitumor activity per modified RECIST NE		Phase II	NCT05673928	2024	[101]
Inotuzumab Ozogamicin	CD22	blinatumomab	older adults with Ph-, CD22+ B-cell ALL	1-year EFS 75%		Phase II	NCT03739814	2023	[105]
		Venetoclax	R/R ALL in Adults		MTD VEN 400 mg/day, InO 0.8/0.5/0.5 mg/m^2^ on days 1, 8 and 15	Phase I	NCT05016947	2023	[104]
Polatuzumab Vedotin	CD79b	Glofitamab, Obinutuzumab	Heavily pre-treated R/R LBCL	ORR 80%, CR rate 62%		Phase Ib Phase II	NCT03533283	2024	[114]
		Lenalidomide, Rituximab	Unfit and frail elderly DLBCL	CR rate 92.9% CR rate100%		Phase II	NCT06176729	2024	[113]
			R/R DLBCL	CR rate 31%		Phase Ib Phase II	NCT02600897	2024	[112]
		Mosunetuzumab SC	Elderly unfit/frail patients with previously untreated DLBCL	ORR 55% (INV-assessed)		Phase Ib Phase II	NCT03677154	2023	[107]
			R/R LBCL	ORR 78%		Phase II	NCT03671018	2024	[106]
			R/R DLBCL	mPFS 11.5 months		Phase III	NCT05171647	2025	[108]
		Mosunetuzumab IV	R/R LBCL	ORR 59.2%		Phase Ib Phase II	NCT03671018	2024	[109]
		FL: Venetoclax, Obinutuzumab DLBCL: Venetoclax, Rituximab	R/R FL or DLBCL	CR rate R/R FL 59.2% R/R DLBCL 31.3%		Phase Ib Phase II	NCT02611323	2024	[110]
		Zanubrutinib, Rituximab	Untreated frail and elderly DLBCL	ORR after 6 cycles 83%		Phase II	NCT05940064	2025	[111]
Enfortumab Vedotin	Nectin-4	Cabozantinib	mUC		Grade ≥ 3 TRAEs 88.9%	Phase I Phase Ib	NCT04878029	2024	[116]
		Erdafitinib	Metastatic bladder cancer		RP2D Erdafitinib 8 mg/day, EV 1.25 mg/kg on days 1, 8 and 15	Phase I	NCT04963153	2025	[115]
Trastuzumab Deruxtecan	HER2	pertuzumab	Previously untreated HER2+ mBC		Grade 3 AEs Diarrhea 6.0%	Phase II	NCT04538742	2024	[118]
		Valemetostat tosylate	Unresectable or metastatic HER2-low BC, a/m HER2+ GC/GEJ adenocarcinoma, a/m nonsquamous NSCLC	Part 2 ORR: Immature	Part 1 safety, tolerability: Immature	Phase I	NCT06244485	2024	[119]
		Zongertinib	mBC and mG/GEJ adenocarcinoma or mGEAC	Phase II ORR: Immature	Phase Ib MTD: Immature	Phase Ib Phase II	NCT06324357	2025	[115]
Sacituzumab Govitecan	Trop-2	Berzosertib	Advanced solid tumors		MTD SG 10 mg/kg, Berzosertib 210 mg/m^2^	Phase I	NCT04826341	2023	[122]
		Enfortumab Vedotin	mUC		MTD SG 10 mg/kg, EV 1.25 mg/kg	Phase I	NCT04724018	2024	[123]
Loncastuximab Tesirine	CD19	Imvotamab	R/R non-HL	ORR: Immature	Safety: Immature	Phase I Phase II	NCT04082936	2023	[126]
		Mosunetuzumab	R/R DLBCL	ORR: Immature	Safety: Immature	Phase II	NCT05672251	2024	[95]
		Arm C: Polatuzumab Vedotin Arm E: Glofitamab Arm F: Mosunetuzumab	R/R DLBCL		Grade 3/4 TRAEs Arm E Arm E 56.1% Arm C, F -	Phase Ib	NCT04970901	2024	[96]
		Rituximab	R/R DLBCL	mPFS 8.3 months		Phase III	NCT04384484	2023	[125]
			R/R FL	CR rate 67%		Phase II	NCT04998669	2025	[124]
Tisotumab Vedotin	TF	Bevacizumab	Recurrent or metastatic CC		DLTs 0% RP2D TV 2 mg/kg, Bevacizumab 15 mg/kg	Phase Ib Phase II	NCT03786081	2023	[42]
Mirvetuximab Soravtansine	FRα	Bevacizumab	Platinum-resistant ovarian cancer	ORR 44%		Phase Ib Phase II	NCT02606305	2023	[127]
			FRα-high platinum-sensitive ovarian cancer	PFS: Immature		Phase III	NCT05445778	2024	[94]
Datopotamab Deruxtecan	Trop-2	Osimertinib	Locally a/m nonsquamous NSCLC	PFS: Immature		Phase III	NCT06350097	2024	[129,130]
			Locally a/m nonsquamous NSCLC	PFS: Immature		Phase III	NCT06417814	2024	[130]
			EGFR-mutated aNSCLC	ORR Dato-DXd 4 mg/kg 43%, Dato-DXd 6 mg/kg 36%		Phase II	NCT03944772	2025	[128]
		Valemetostat tosylate	a/mNSCLC	Part 2 ORR: Immature	Part 1 safety, tolerability: Immature	Phase Ib	NCT06244485	2025	[120,132]
Telisotuzumab Vedotin	c-Met	Osimertinib	c-Met protein-overexpressing, EGFR-mutated locally a/mNSCLC after progression on prior osimertinib	ORR 50%, mPFS 7.4 months, DOR NR	Grade ≥ 3 AEs 50%	Phase I Phase Ib	NCT02099058	2025	[93]

a/m: advanced or metastatic, a/mNSCLC: advanced or metastatic non-small-cell lung cancer, AEs: adverse events, ALL: acute lymphoblastic leukemia, aNSCLC: advanced non-small-cell lung cancer, BC: breast cancer, CC: cervical cancer, CNS: central nervous system, CR: complete response, Dato-DXd: Datopotamab Deruxtecan, DLBCL: diffuse large B-cell lymphoma, DLTs: dose-limiting toxicities, DOR: duration of response, EFS: event-free survival, EV: Enfortumab vedotin, FL: follicular lymphoma, GC/GEJ: gastric cancer or gastroesophageal junction, HER2+: HER2-positive, HL: Hodgkin lymphoma, Immature: data not yet mature for analysis, InO: inotuzumab ozogamicin, INV: investigator-assessed, IV: intravenous, LBCL: large B-cell lymphoma, mBC: metastatic breast cancer, mG/GEJ: metastatic gastric or gastroesophageal junction, mGEAC: metastatic gastroesophageal adenocarcinoma, mOS: median overall survival, mPFS: median progression free survival, MTD: maximum tolerated dose, mUC: metastatic urothelial carcinoma, NR: not reached, NSCLC: non-small-cell lung cancer, ORR: objective response rates, Ph-: Philadelphia chromosome-negative, R/R: relapsed or refractory, RP2D: recommended phase II dose, SC: subcutaneous, SG: sacituzumab govitecan, TRAEs: treatment-related adverse events, TV: tisotumab vedotin, VEN: venetoclax.

### 2.2. Recent Multi-Modal Combination Therapies

#### 2.2.1. ADC + Chemotherapy + ICIs

Triple combination therapy with ADCs, ICIs, and chemotherapy has recently emerged as a promising strategy to improve clinical outcomes in patients with advanced or metastatic (a/m) cancers. This approach holds the potential to overcome the limitations of current standard treatments and achieve superior therapeutic efficacy. ADCs selectively target cancer cells, ICIs enhance antitumor immune responses by activating the immune system, and chemotherapy not only destroys tumor cells but also induces immunogenic cell death. These complementary mechanisms enable multi-target and synergistic effects, reduce the likelihood of resistance development, and enhance both the intensity and durability of treatment responses. Furthermore, ADCs targeting antigens such as Trophoblast cell surface antigen 2 (TROP2) or Human Epidermal Growth Factor Receptor 2 (HER2) may expand the therapeutic benefit to a broader patient population when combined with ICIs and chemotherapy. Ongoing clinical trials are actively investigating this triple combination strategy in various malignancies, including NSCLC, gastric or gastroesophageal adenocarcinoma/gastroesophageal junction cancer (GEA/GEJC), and cHL.

***Brentuximab vedotin***

For an extended period, ABVD (doxorubicin, bleomycin, vinblastine, dacarbazine) was the standard first-line treatment for advanced cHL in North America. However, due to significant toxicity concerns, the A + AVD regimen (BV, doxorubicin, vinblastine, dacarbazine), which includes BV, was approved as a new standard of care based on the results of the ECHELON-1 trial. Nonetheless, A + AVD was associated with a higher incidence of peripheral neuropathy and febrile neutropenia, likely due to overlapping mechanisms of action between BV and vinblastine.

In a phase II clinical trial enrolling 58 patients, it was hypothesized that replacing vinblastine with nivolumab would improve the regimen’s efficacy and tolerability. To test this, they investigated the AN + AD (BV, nivolumab, doxorubicin, dacarbazine) combination in treatment-naïve patients with stage II bulky or stage III/IV cHL. At the end of treatment (EOT), patients treated with AN + AD achieved a CR rate of 88% and a 2-year PFS rate of 88%, both higher than those reported for the A + AVD arm in the ECHELON 1 trial (73% and 82%, respectively). In this regard AN + AD showed a positive safety profile, with no febrile neutropenia (0%) and grade ≥3 peripheral neuropathy in only 4% of patients, compared with 19% and 11% in ECHELON 1 and 7% and 9% in SWOG S1826, respectively. These findings suggest that AN + AD not only offers improved efficacy over A + AVD but also provides a favorable safety profile [133].

***Trastuzumab deruxtecan***

Trastuzumab deruxtecan (T-DXd) is a HER2-targeted ADC approved for the treatment of previously treated HER2-positive (HER2+) metastatic gastric adenocarcinoma or GEJA, metastatic NSCLC with activating HER2 mutations, HER2+ breast cancer, and HER2-low breast cancer. Preclinical studies have demonstrated that combining T-DXd with immunotherapy yields superior antitumor activity compared with monotherapy, and that the addition of chemotherapy may further enhance efficacy. Based on this evidence, multiple clinical trials are actively investigating T-DXd-based combination regimens.

The DESTINY-Gastric03 (DG-03) trial is a multicenter, open-label, phase Ib/II study designed to evaluate the safety and efficacy of T-DXd monotherapy and combination regimens in patients with HER2-expressing (HER2+ and HER2-low) unresectable, locally advanced, or metastatic gastric cancer, GEJA, or esophageal adenocarcinoma.

Janjigian, Raoufmoghaddam et al. (2024) reported the DG-03 trial design, which enrolled treatment-naïve patients with HER2+ or HER2-low gastric cancer (including GEJA and esophageal adenocarcinoma). Part 2 evaluated T-DXd in combination with a fluoropyrimidine (5-fluorouracil (5-FU) or capecitabine (CAPE)) and pembrolizumab in previously untreated HER2+ gastric cancer. Part 3 evaluated T-DXd plus a fluoropyrimidine and volrustomig, a Programmed cell death protein-1 (PD-1)/cytotoxic T-lymphocyte antigen-4 (CTLA-4) bispecific antibody, in previously untreated HER2+ or HER2-low gastric cancer. These regimens were designed to combine the HER2-targeted activity of T-DXd, the cytotoxic effects of chemotherapy, and the immune-activating effects of ICIs to assess safety and efficacy in the first-line setting, and the trial is currently ongoing [134].

Janjigian et al. (2024) described Part 4, which was added in a November 2023 protocol amendment. Part 4 evaluates T-DXd 5.4 mg/kg in combination with rilvegostomig, a PD-1/T-cell immunoreceptor with Ig and ITIM domains (TIGIT) bispecific antibody, and a fluoropyrimidine (5-FU or CAPE) in patients with HER2+ or HER2-low gastric, GEJA, or esophageal adenocarcinoma. Rilvegostomig targets both PD-1 and TIGIT pathways to enhance antitumor immune responses. This triplet regimen, combining a HER2-targeted ADC, an ICI, and cytotoxic chemotherapy, aims to achieve improved antitumor efficacy and disease control through multiple complementary mechanisms of action. This part of the trial is also ongoing [135].

Janjigian et al. (2025) presented updated Part 2 results from treatment-naïve patients with HER2+ gastric cancer, GEJA, or esophageal adenocarcinoma receiving T-DXd, pembrolizumab, and a fluoropyrimidine (5-FU or CAPE). This analysis compared two dose levels of T DXd: 6.4 mg/kg (Arm D) and 5.4 mg/kg (Arm F), with the confirmed ORR being 53.5% and 75.0%, respectively, and particularly high responses observed in the lower dose arm among IHC 3+ (73.1%) and IHC 2+/ISH+ (83.3%) subgroups. Notably, while the FDA approved dose of T DXd for HER2+ gastric cancer, GEJA monotherapy is 6.4 mg/kg, these results suggest that in combination with pembrolizumab and chemotherapy, a reduced dose of 5.4 mg/kg can achieve a higher confirmed ORR, potentially enhancing tolerability without compromising efficacy. These results are based on data with a 6 May 2024, data cutoff, and the trial is ongoing [136,137].

The ongoing DESTINY-Gastric05 phase III trial has enrolled 576 patients with HER2+ gastric or GEJ cancer and is evaluating T-DXd in combination with pembrolizumab and a fluoropyrimidine (5-FU or CAPE). This regimen is being compared with standard-of-care trastuzumab plus platinum-based chemotherapy and pembrolizumab, with the goal of establishing a platinum-free treatment approach with potential mechanistic advantages for all HER2+ patients. The primary endpoint is PFS, and results have not yet been reported [138].

In the lung oncology setting, T-DXd combination strategies are also under active investigation. The DESTINY Lung03 trial is an open label, multicenter, phase Ib, multipart study evaluating the safety and efficacy of T DXd combined with an ICI and chemotherapy in the first line treatment of non-squamous NSCLC with HER2 overexpression. A total of 148 patients are planned for enrollment across all parts of the trial. In Part 1, durvalumab was combined with cisplatin, carboplatin, or pemetrexed; in Part 3, the PD-1/CTLA-4 bispecific antibody MEDI5752 was combined with carboplatin; and in Part 4, T DXd is being tested with rilvegostomig, a PD 1/TIGIT bispecific antibody, with or without carboplatin, in previously untreated HER2 overexpressing NSCLC. Preclinical data demonstrated that combining the DXd payload of T-DXd with an ICI produced superior antitumor activity compared with monotherapy, and that adding chemotherapy could further enhance efficacy. The trial remains ongoing, and updated efficacy and safety results from the combination arms have not yet been reported [139,140].

***Datopotamab deruxtecan***

Dato-DXd, a novel ADC targeting TROP2 protein, has recently emerged as a promising therapeutic agent in oncology. Multiple clinical trials are currently evaluating Dato-DXd in combination with other anticancer agents to enhance efficacy across various tumor types.

The phase I TROPION-Lung02 trial is a multicohort, first-line study evaluating Dato-DXd in combination with pembrolizumab with or without platinum-based chemotherapy (cisplatin or carboplatin) in patients with a/mNSCLC. Among 96 patients included in the pooled analysis of all six cohorts, the triplet regimen demonstrated an ORR of 56%, indicating durable antitumor activity. Notably, stomatitis, one of the most common treatment-related adverse events, occurred in 33% of patients receiving the triplet therapy, a lower incidence compared to 57% observed in the dual combination of Dato-DXd and pembrolizumab. These findings suggest that the addition of chemotherapy did not exacerbate toxicity and that the overall safety profile of the triplet regimen remains manageable [141,142].

Building on the favorable findings from TROPION-Lung02, the TROPION-Lung07 study, which is currently ongoing with 1170 patients enrolled, was initiated as a phase III clinical trial to evaluate Dato-DXd combination therapy as a first-line treatment option for patients with non-squamous a/mNSCLC who lack actionable genomic alterations and have low PD-L1 expression (tumor proportion score [TPS] < 50%). It is well established that in such patients, clinical outcomes tend to be inferior compared with those in patients with high PD-L1 expression, and treatment-related toxicities may be more challenging to manage. Consequently, there is a strong need to explore novel therapeutic strategies that could improve efficacy in this patient population. In TROPION-Lung07, the experimental regimen consists of Dato-DXd plus pembrolizumab combined with platinum-based chemotherapy. This approach aims to leverage the complementary mechanisms of action of a TROP2-targeted ADC, an ICI, and cytotoxic chemotherapy to maximize antitumor efficacy. The primary endpoints are PFS and OS, but results have not yet been reported as the study is still ongoing [143].

In patients with a/mNSCLC, the combination of immunotherapy and chemotherapy has shown limited improvement in 5-year OS. The AVANZAR trial is a phase III clinical study designed to assess a novel treatment strategy by combining the TROP2-targeted ADC Dato-DXd with the anti–programmed death-ligand 1 (PD-L1) immunotherapy durvalumab and carboplatin. The primary endpoints are PFS and OS assessed by blinded independent central review (BICR). The trial is currently ongoing, and results for the primary endpoints have not yet been reported [144].

Similarly, the TROPION-Lung04 trial, a phase Ib study, also assessed this triplet combination as a first-line option in the same group of patients, with safety as the primary endpoint. A total of 37 patients in cohort 4 were enrolled and treated with Dato-DXd and durvalumab in combination with carboplatin. The safety profile of Dato-DXd was consistent with previous reports, with no newly identified serious adverse events. In cohort 4, the ORR was 56.8%, the mDoR was 8.8 months, and the safety was considered acceptable. These results suggest that Dato-DXd-based combination therapy may offer manageable clinical benefit with an acceptable safety profile in a/mNSCLC [145].

#### 2.2.2. ADC + Chemotherapy + Molecular Targeted Cancer Therapy

The combination of ADCs and molecular targeted anticancer agents with conventional chemotherapy is emerging as a transformative strategy in oncology. Such combinatorial approaches offer enhanced antitumor activity along with favorable safety profiles. Notably, a variety of ADCs deliver cytotoxic payloads selectively to tumor-specific targets, thereby minimizing toxicity to normal tissues and reducing the systemic adverse effects commonly associated with chemotherapy. A growing number of next-generation clinical trials are actively investigating these multi-mechanistic strategies, continuously generating promising and clinically meaningful outcomes that are reshaping the landscape of cancer treatment.

***Gemtuzumab ozogamicin***

As previously described, GO is a CD33-targeted ADC approved for use as a standalone therapy or in combination with chemotherapy in patients with CD33-positive AML [146].

Recent clinical studies have demonstrated that CD33 is expressed at relatively high levels on leukemic blasts in patients with FLT3-mutated AML, providing a biological rationale for the integration of GO into existing treatment regimens. Based on this rationale, ongoing clinical trials are investigating the safety and efficacy of combining GO with cytarabine and anthracyclines, which comprise the standard induction regimen, along with midostaurin, a FLT3 inhibitor. Notably, the phase I clinical trial, which enrolled 21 patients, reported promising complete remission rates and a manageable toxicity profile in newly diagnosed (ND) FLT3-mutated AML patients treated with GO and midostaurin in combination with the 7 + 3 regimen (cytarabine and daunorubicin). Toxicities were similar to those observed with standard intensive induction therapy. The OS rates were 79% at 6 months and 65% at 1 year, and the addition of GO and midostaurin to standard therapy appeared to be well tolerated in this population [147].

Furthermore, the SAL-MODULE study, a phase I trial, extended this combination strategy to patients with CBF-AML and FLT3-mutated AML, demonstrating its broader applicability and clinical feasibility. This phase I study was primarily conducted to establish the maximum tolerated dose (MTD) and the RP2D, and confirmed that the combination of GO and midostaurin can be safely administered with intensive chemotherapy in newly diagnosed AML patients. The ORR was remarkably high at 92%, including a complete remission rate of 75%. In addition, the 2-year RFS and OS rates were 81% and 100%, respectively, further supporting the safety and clinical potential of this regimen [148].

***Inotuzumab ozogamicin***

InO is an ADC targeting CD22, which has demonstrated potent antitumor activity in R/R ALL. Recently, in an effort to overcome the limitations of monotherapy, combination strategies with other molecularly targeted agents and low-intensity chemotherapy have been actively investigated.

In one phase II study involving newly diagnosed Philadelphia chromosome-positive (Ph+) ALL patients, a triplet regimen was employed, combining dual-targeted induction therapy with dasatinib (DAS) and InO followed by maintenance chemotherapy with POMP (6-mercaptopurine, vincristine, methotrexate, and prednisone). This approach resulted in deep and sustained molecular responses. At the end of course 2, which included only DAS and InO, 61% of patients had achieved CMR and 22% had achieved major molecular response (MMR), indicating the initial effectiveness of the induction regimen. However, the addition of POMP during the maintenance phase further improved treatment outcomes: by the end of course 3, all 18 patients had achieved either CMR as determined by qRT-PCR and/or MRD negativity by next-generation sequencing (NGS). The CMR rate increased to 89%, and MRD negativity rose from 67% at the end of course 2 to 89% after course 3. These findings underscore the enhanced efficacy of the triplet approach in achieving deeper molecular remission beyond MMR [149].

In another clinical study, the efficacy and safety of a treatment regimen consisting of mini-Hyper-CVD (dose-reduced cyclophosphamide, vincristine, and dexamethasone) in combination with a reduced and fractionated dose InO, followed by sequential administration of blinatumomab, were evaluated in patients with R/R B-ALL. This phase II trial, conducted up to February 2024, enrolled 133 patients and was divided into three cohorts. Cohort 1 received mini-Hyper-CVD plus InO alone; Cohort 2 included sequential blinatumomab; the dose-dense (d-d) cohort received all three agents concurrently from the outset. The ORR improved markedly across cohorts—from 76% in Cohort 1, to 93% in Cohort 2 with sequential blinatumomab, and up to 100% in the dose-dense cohort. MRD negativity rates also increased from 82% in Cohort 1 to 85% in Cohort 2 and 95% in the dose-dense cohort. Notably, the addition of blinatumomab not only enhanced MRD responses but also increased the interval between the last dose of InO and allogeneic SCT, helping to reduce safety-related complications such as hepatic SOS. These results strongly suggest that, for patients with R/R B-ALL, a triplet regimen consisting of mini-Hyper-CVD, InO, and blinatumomab, particularly when blinatumomab is given early and intensively, can lead to very deep and rapid MRD responses and outstanding overall therapeutic outcomes [150,151,152].

Another example is a phase II trial that evaluated hyper-CVAD (hyperfractionated cyclophosphamide, vincristine, doxorubicin, and dexamethasone) with blinatumomab and InO in patients with newly diagnosed Ph-negative B-cell ALL. Among the 69 patients enrolled, 31 received the triplet regimen including InO, with a 15-month OS rate of 100% compared to 87% in the non-InO group. No treatment discontinuations due to InO-related toxicities occurred, and no cases of sinusoidal obstruction syndrome/veno-occlusive disease (SOS/VOD) were observed. These findings suggest that adding InO to hyper-CVAD and sequential blinatumomab is safe and may improve survival in patients with newly diagnosed Ph-negative B-cell ALL [153,154].

***Polatuzumab vedotin***

Pola, an ADC targeting CD79b, has been utilized to address the limitations of the R-CHOP (Rituximab, Cyclophosphamide, Doxorubicin, Vincristine, and Prednisone) regimen by aiming to improve safety and efficacy.

On 19 April 2023, FDA approved the Pola-R-CHP (Polatuzumab vedotin, Rituximab, Cyclophosphamide, Doxorubicin Hydrochloride, Prednisone) regimen as a first-line treatment for previously untreated DLBCL or high-grade B-cell lymphoma (HGBL), based on the results of the phase III POLARIX trial. This approval was driven by a significant improvement in PFS compared to standard R-CHOP therapy. The phase III POLARIX trial, which evaluated the Pola-R-CHP regimen, established it as a new standard of care. In the final analysis as of 5 July 2024, the 5-year PFS rate for the Pola-R-CHP group were 64.2% in the global ITT population (*n* = 879) and 63.1% in the expanded population (*n* = 1000), compared to 59.1% in both populations for the R-CHOP group, demonstrating a meaningful improvement. Consequently, there has been growing interest in exploring Pola-based combination therapies for high-risk subtypes of DLBCL and R/R patient populations [155,156].

Additionally, while the POLARIX study showed Pola was more effective than vincristine in R-CHOP, its safe integration into intensified regimens like DA-EPOCH-R for high-risk lymphoma subtypes remained unclear. To explore this, a phase I study replaced vincristine with Pola in the DA-EPOCH-R regimen, resulting in the Pola-DA-EPCH-R combination. The primary objective was to assess safety based on DLTs during the first two cycles; 3 of 18 patients experienced DLTs, meeting the predefined safety threshold. Replacing vincristine with Pola did not impair dose escalation and was feasible in subtypes requiring intensified therapy and Pola’s benefits. It also reduced peripheral neuropathy while maintaining efficacy, broadening Pola’s applicability in aggressive large B-cell lymphomas [157].

In the phase Ib BO42203 trial, the addition of the oral BCL-2 inhibitor venetoclax (Ven) to the Pola-R-CHP regimen was evaluated in treatment-naïve, high-risk BCL-2 IHC-positive DLBCL patients, including those with double-hit and triple-hit lymphomas (DHL/THL). A total of 50 patients were enrolled. No DLTs, the primary safety endpoint, were observed. The PET-CT-based ORR was 86.0%, and the CR rate was 82.0%, with particularly high CR rates observed across all cohorts, including in patients with DHL/THL. This combination demonstrated strong antitumor activity with an acceptable safety profile and no new safety signals. Although promising, the combination of venetoclax and Pola-R-CHP remains investigational and is still under clinical evaluation [158].

The phase III clinical trial, known as POLAR BEAR, compares R-mini-CHOP with R-mini-CHP, which includes Pola, primarily in elderly patients with DLBCL. A total of 200 participants are expected to be enrolled. The primary endpoint, similar to that of the POLARIX trial, is PFS and the trial is currently ongoing [159].

The combination of glofitamab, a CD20xCD3 bispecific antibody, with Pola in the Pola-R-CHP backbone has emerged as a promising first-line treatment strategy for DLBCL. In the phase Ib study, among 80 previously untreated DLBCL patients, 24 were enrolled in the glofitamab-Pola-R-CHP arm, which achieved an ORR of 100% and a CMR rate of 96%, with a manageable safety profile. Similarly, in the phase II COALITION study, 40 of 80 high-risk DLBCL patients aged ≤65 years received glofitamab-Pola-R-CHP, with over 95% completing all planned therapy and achieving ORR and CR rate of 100% and 98% at the end of induction. Given the promising efficacy and safety observed in these earlier studies, the ongoing global phase III SKYGLO trial is expected to definitively evaluate the integration of a bispecific antibody into standard chemoimmunotherapy. This study will enroll approximately 1130 treatment-naïve DLBCL patients aged 18 to 80 years and compare glofitamab-Pola-R-CHP with Pola-R-CHP, with PFS as the primary endpoint [160,161,162].

Beyond these settings, Pola has also shown clinical benefit in other therapeutic contexts. In the POLAROSE phase III trial involving transplant-ineligible Chinese patients with R/R DLBCL, the Pola plus bendamustine and rituximab (Pola-BR) regimen demonstrated a significant clinical advantage over BR alone. Specifically, the Pola-BR group had a 10.7% higher CR rate (25.0% vs. 14.3%), with PFS extended by 2.6 months and OS by 4.1 months. The risk of progression or death was reduced by 50%, and the risk of overall mortality by 45%. These findings support Pola as a versatile agent capable of enhancing outcomes across various treatment settings by overcoming the limitations of conventional chemotherapy and improving survival in patients with limited treatment options for DLBCL [163].

***Loncastuximab tesirine***

Loncastuximab tesirine (LT) is a CD19-targeting ADC proposed as a combinatorial strategy to address the limitations of the standard R-CHOP regimen in DLBCL.

This phase Ib clinical study evaluates the LoRR-CHOP regimen, which combines LT and the phosphodiesterase-4 (PDE4) inhibitor roflumilast with R-CHOP. The study focuses on assessing the safety, tolerability, and antitumor activity of this combination. LT targets CD19-expressing cells with a potent cytotoxin, while roflumilast inhibits PDE4, which is overexpressed in the non-germinal center B-cell-like (GCB) subtype and linked to poor prognosis, to disrupt key survival pathways in DLBCL, together impairing tumor cell growth and survival. Given the complementary mechanisms of action of each agent, the LoRR-CHOP regimen is currently being evaluated for its potential as a novel immunochemotherapy-based treatment strategy. The trial is actively recruiting patients, and preliminary safety and efficacy data are expected to be reported [164].

***Mirvetuximab soravtansine***

MIRV is an ADC that selectively binds to FRα, enabling targeted cytotoxicity against FRα-overexpressing EOC cells. EOC is the most lethal gynecologic malignancy, and treatment of recurrent disease remains challenging due to the cumulative toxicities of conventional chemotherapy, such as peripheral neuropathy and myelosuppression, which often limit treatment continuity. To address these limitations, combination strategies incorporating MIRV have garnered increasing attention.

In this context, the phase Ib FORWARD II trial evaluated a triplet regimen of MIRV, carboplatin, and bevacizumab in patients with recurrent, platinum-sensitive EOC. Among 41 enrolled participants, the triplet regimen exhibited an adverse event profile in line with the known safety of the individual agents, and no new safety signals were detected. Peripheral neuropathy was manageable in this setting. The confirmed ORR was 83%, and notably, 40 of 41 patients experienced a reduction in target lesion size. While higher FRα expression has been linked to greater responses with MIRV monotherapy, the results of this study suggest that the triplet regimen could offer therapeutic advantages across a broader patient population. Such outcomes indicate that this approach has the potential to serve as an effective alternative to taxane-based regimens, with a safety profile consistent with that of the individual agents while utilizing FRα-targeted therapy for precise tumor control [165].

#### 2.2.3. ADC + ICIs + Molecular Targeted Cancer Therapy + Chemotherapy

***Brentuximab vedotin***

BV, an ADC targeting CD30, has emerged as a therapeutic option for primary mediastinal large B-cell lymphoma (PMBL). This disease is characterized by distinctive molecular features, including CD30 expression, amplification of the 9p24.1 locus, and overexpression of PD-1 ligands. Based on these biological characteristics, the combination of BV with the ICI nivolumab has demonstrated clinically meaningful responses in patients with R/R PMBL.

Building upon this therapeutic potential, an ongoing phase II clinical trial is currently evaluating a quadruplet regimen that adds apoptosis-inducing agents including R-CHP (rituximab, cyclophosphamide, doxorubicin hydrochloride, prednisone) to the BV and nivolumab backbone, as a first-line treatment for previously untreated PMBL patients. This study hypothesizes that the quadruplet combination may achieve similarly high ORR in the first-line setting as observed in the R/R PMBL population, while avoiding chemoresistance, reducing the intensity of chemoimmunotherapy (CIT), and minimizing the necessity for consolidative radiotherapy (XRT). This ongoing trial aims to enroll 40 patients and evaluates the CR rate at the EOT as its primary endpoint. Given that most patients with PMBL respond well to initial therapy but have poor prognoses upon relapse or resistance, the development of novel treatment strategies may represent a significant advancement in the management of this disease [166].

Table 5 summarizes multiplet ADC-based combination therapies in clinical trials.

**Table 5:** Summary of multiplet ADC-based combination therapies in clinical trials. This table provides an overview of partner drugs, indications, primary efficacy and safety outcomes, clinical trial details, and publication year.

ADC	Target	Partner Drug Type	Pertner Drug	Indication	Primary Endpoint	Phase	NCT Number	Publication Year	Ref.
Gemtuzumab Ozogamicin	CD33	Molecular targeted cancer therapy + Chemotherapy	Midostaurin, Cytarabine, Daunorubicin	FLT3- mutated AML, CBF leukemia	MTD Cytarabine 200 mg/m^2^/day (Day 1–7), Daunorubicin 60 mg/m^2^/day (Day 1–3), GO 3 mg/m^2^ (Days 1 and 4), Midostaurin 100 mg/day (Day 8–21)	Phase I Phase II	NCT04385290	2024	[148]
			Midostaurin, Cytarabine, Daunorubicin	FLT-3 Mutated AML	DLT DL1, DL4 0% DL3 33.3% DL2 11.1%	Phase I	NCT03900949	2024	[147]
Brentuximab Vedotin	CD30	ICI + Chemotherapy	Nivolumab, AD	Advanced-stage cHL	CR rate 88%	Phase II	NCT03646123	2025	[133]
		ICI + Molecular targeted cancer therapy + Chemotherapy	Nivolumab, R-CHP	PMBL	CR rate: Immature	Phase II	NCT04745949	2023	[166]
Inotuzumab Ozogamicin	CD22	Molecular targeted cancer therapy + Chemotherapy	hyper-CVAD, Blinatumomab	Newly diagnosed Ph- B-cell ALL	3-year OS 87%	Phase II	NCT02877303	2023	[153,154]
			Mini-hyper-CVD, Blinatumomab	R/R B-cell ALL	ORR cohort1 76%, cohort2 93%, cohort D-D 100% MRD negativity cohort1 82%, cohort 2 85%, cohort D-D 95% 1-year OS cohort1 51%, cohort2 66%, cohort D-D 90%	Phase I Phase II	NCT01371630	2024	[151]
			Dasatinib, Dexamethasone, POMP	Ph+ ALL	CMR rate 89%	Phase II	NCT04747912	2024	[149]
Polatuzumab Vedotin	CD79b	Molecular targeted cancer therapy + Chemotherapy	DA-EPCH-R	Aggressive B-cell non-HL	DLT 16.7%	Phase I	NCT04231877	2023	[157]
			R-CHP	Intermediate- or high-risk DLBCL	5-year PFS In the global ITT population 64.2% In the expanded population 63.1%	Phase III	NCT03274492	2024	[155]
			R-mini-CHP	Elderly patients with previously untreated DLBCL	PFS: Immature	Phase III	NCT04332822	2024	[159]
			Venetoclax, R-CHP	Untreated high-risk BCL-2-Positive B-cell lymphoma	DLTs 0% RP2D Ven 800 mg/D 5 days/cycle	Phase I	NCT04790903	2024	[158]
			Rituximab, Bendamustine	R/R DLBCL	CR rate 25%	Phase III	NCT04236141	2024	[163]
			Rituximab, Bendamustine Obinutuzumab, Bendamustine	R/R FL	CR rate Rituximab, Bendamustine 68% Obinutuzumab, Bendamustine 70%	Phase I Phase II	NCT02257567	2023	[167]
			Glofitamab, R-CHP	Previously untreated CD20+ LBCL	PFS: Immature	Phase III	NCT06047080	2024	[162]
				previously untreated DLBCL	ORR 100%, CMR rate 96% Grade ≥ 3 AEs 67% neutropenia 58%, febrile neutropenia 8%, thrombocytopenia 17%. anemia 17%	Phase Ib	NCT03467373	2025	[160]
				Younger patients with high-risk LBCL	Grade ≥ 3 AEs 58%	Phase II	NCT04914741	2025	[161]
Trastuzumab Deruxtecan	HER2	ICI + Chemotherapy	Nivolumab, Capecitabine, Oxaliplatin	HER2-low expressing gastroesophageal adenocarcinoma	Phsae Ib DLTs: Immature Phase II ORR: Immature	Phase Ib Phase II	jRCT2031230477	2024	[168]
			Part 1: - Durvalumab, Cisplatin - Durvalumab, Carboplatin - Durvalumab, Pemetrxed Part 3: MEDI5752, Carboplatin Part 4: Rilvegostomig, carboplatin	a/m nonsquamous NSCLC and HER2 overexpression	Safety: Immature	Phase I	NCT04686305	2023	[139,169]
			Rilvegostomig, 5-Fluorouracil or Capecitabine	HER2+ and HER2-low gastric or GEJ adenocarcinoma	ORR: Immature	Phase II	NCT04379596	2024	[135]
			Arm 2F Pembrolizumab, 5-Fluorouracil or Capecitabine Arm 3A, 3B Volrustoming, 5-Fluorouracil or Capecitabine	HER2+ and HER2-low gastric cancer	ORR: Immature	Phase II	NCT04379596	2024	[134]
			Pembrolizumab + 5-Fluorouracil or Capecitabine	a/m HER2+ gastric cancer, GEJ adenocarcinoma, or esophageal adenocarcinoma	ORR Arm 2D (DCO 15 February 2023) 55.9% Arm 2F (DCO 6 May 2024) 73.1%	Phase II	NCT04379596	2025	[136]
				Metastatic HER2+ gastric or GEJ cancer	PFS: Immature	Phase III	NCT06731478	2025	[138]
Loncastuximab Tesirine	CD19	Molecular targeted cancer therapy + Chemotherapy	Roflumilast, R-CHOP	Naïve high-risk DLBCL	Safety: Immature	Phase I	NCT02606305	2025	[164]
Mirvetuximab Soravtansine	FRα	Molecular targeted cancer therapy + Chemotherapy	Carboplatin, Bevacizumab	Platinum-sensitive ovarian cancer	ORR 83%	Phase I Phase II	NCT04526691	2024	[165]
Datopotamab Deruxtecan	Trop-2	ICI + Chemotherapy	Durvalumab, Carboplatin	a/mNSCLC	Grade ≥ 3 TEAEs 65.4%	Phase I	NCT04612751	2025	[145]
				a/mNSCLC	PFS, OS: Immature	Phase III	NCT05687266	2023	[144]
			Pembrolizumab, Carboplatin, Cisplatin	aNSCLC	Serious TRAEs 22%	Phase I	NCT04526691	2025	[141]
				aNSCLC	PFS, OS: Immature	Phase III	NCT05555732	2024	[143]

AML: acute myeloid leukemia, a/m: advanced or metastatic, a/mNSCLC: advanced or metastatic non-small-cell lung cancer, AD: doxorubicin, dacarbazine, AEs: adverse events, ALL: acute lymphoblastic leukemia, aNSCLC: advanced non-small-cell lung cancer, CBF: core-binding factor, cHL: classical Hodgkin lymphoma, CMR: complete molecular response, CR: complete response, CVAD: cyclophosphamide, vincristine, doxorubicin, dacarbazine, CVD: cyclophosphamide, vincristine, dacarbazine, DA-EPCH-R: rituximab, etoposide, prednisone, cyclophosphamide, doxorubicin, DCO: data cutoff, D-D: dose-dense, DLBCL: diffuse large B-cell lymphoma, DLTs: dose-limiting toxicities, FL: follicular lymphoma, GEJ: gastroesophageal junction, GO: gemtuzumab ozogamicin, HER2+: HER2-positive, HL: Hodgkin lymphoma, ICI: immune checkpoint inhibitor, Immature: data not yet mature for analysis, ITT: intent-to-treat, LBCL: large B-cell lymphoma, MRD: minimal residual disease, MTD: maximum tolerated dose, ORR: objective response rates, OS: overall survival, PFS: progression free survival, Ph-: Philadelphia-chromosome negative, Ph+: Philadelphia chromosome-positive, PMBL: primary mediastinal large B-cell lymphoma, POMP: 6-mercaptopurine, vincristine, methotrexate, and prednisone, R/R: relapsed or refractory, R-CHOP: rituximab, cyclophosphamide, doxorubicin, vincristine, prednisone, R-CHP: rituximab, cyclophosphamide, doxorubicin, prednisone, RP2D: recommended phase II dose, TRAEs: treatment-related adverse events, Ven: venetoclax.

## 3. Conclusions

With advances in cancer therapeutics, ADCs have emerged as a transformative approach in oncology, offering robust efficacy and favorable safety profiles even as monotherapy. Their ability to selectively target tumor-associated antigens while minimizing off-target toxicity has positioned them as a new standard in clinical practice.

Despite this success, challenges such as resistance, antigen heterogeneity, and limited durability have highlighted the need for combination strategies. Accordingly, growing interest has centered on pairing ADCs with chemotherapeutics, ICIs, molecularly targeted agents, and even triplet regimens to enhance both efficacy and tolerability. Although not covered in this review, combination strategies such as ADC–ADC, ADC–radiotherapy, and ADC–cell therapy approaches are also being actively investigated in clinical settings. Numerous clinical trials across various cancers are already showing promising results. These combination approaches may improve response rates, reduce toxicity, and enable tailored options for older or clinically vulnerable patients.

However, several clinical challenges remain, including toxicity-management issues, optimal dose scheduling, and the identification of patient populations most likely to benefit from specific combinations. In addition, limited understanding of resistance mechanisms and the absence of validated biomarkers continue to hinder broader clinical translation of ADC-based combinations. Looking ahead, ADC-based regimens are expected to evolve through biomarker-driven patient selection, optimized combinations, and refined safety management. Future research should prioritize the integration of translational studies, rational design of synergistic combinations, and adaptive trial frameworks to overcome current limitations and fully realize the potential of ADC-based multi-modal therapy. ADCs, whether in monotherapy or combination therapy, are expected to assume an important role in the evolving field of precision cancer therapy.

## Figures and Tables

**Table 1:** Summary of FDA-approved antibody–drug conjugates (ADCs) [1]. This table provides an overview of the structural components (target antigen, antibody, isotype, linker, payload, and DAR) and key clinical features (indications, approval details, and pivotal trials) of approved ADCs.

ADC (Brand Name)	Target	Antibody	Isotype	Linker	Payload	DAR	Indication	Indication Details (Approval Date)	Pivotal Trials (Phase)	Ref.
Gemtuzumab Ozogamicin (Mylotarg)	CD33	Gemtuzumab	IgG4κ	AcButDMH	Calicheamicin	2–3	AML	Newly diagnosed CD33+ AML in adults and pediatric patients, R/R CD33+ AML in adults and pediatric patients aged 2 years and older (22 February 2000; Withdrawn: 28 November 2011; Re-approved: 1 September 2017)	ALFA-0701 (Phase III)	[7]
								Newly diagnosed CD33+ AML in pediatric ≥ 1 month (16 June 2020)	AAML0531 (Phase III)	[7,8]
Brentuximab Vedotin (Adcetris)	CD30	Brentuximab	IgG1κ	mc-VC-PABC	MMAE	4	HL, ALCL	HL in patients who have relapsed after ASCT, or after failure of ≥2 prior multi-agent chemotherapy regimens in those ineligibles for ASCT, sALCL following failure of at least one prior multi-agent chemotherapy regimen (19 August 2011)	SGN35-0003 (Phase II) SGN35-0004 (Phase II)	[7,9,10]
							pcALCL, MF	pcALCL or CD30-expressing MF in adults after prior systemic therapy (9 November 2017)	ALCANZA (Phase III)	[7,11,12]
Trastuzumab Emtansine (Kadcyla)	HER2	Trastuzumab	IgG1κ	SMCC	DM1	3.5	BC	HER2+ mBC with prior exposure to trastuzumab and a taxane, with prior therapy for metastatic disease or recurrence within 6 months of completing adjuvant therapy (22 February 2013)	EMILIA (Phase III)	[13]
								HER2+ EBC with residual disease after neoadjuvant taxane- and trastuzumab-based therapy (3 May 2019)	KATHERINE (Phase III)	[14]
Inotuzumab Ozogamicin (Besponsa)	CD22	Inotuzumab	IgG4κ	AcButDMH	Calicheamicin	6	B-cell precursor ALL	R/R CD22+ B-cell precursor ALL in adults (17 August 2017)	INO-VATE (Phase III)	[15]
								R/R CD22+ B-cell precursor ALL in pediatric ≥ 1 year (6 March 2024)	ITCC-059 (Phase II)	[16]
Moxetumomab Pasudotox (Lumoxiti)	CD22	Moxetumomab	IgG4κ	mc–VC–PABC	PE38	1.8	HCL	R/R in adults after ≥2 prior systemic therapies including a purine nucleoside analog (13 September 2018; voluntary market withdrawn: August 2023)	Study 1053 (Phase III)	[17]
Polatuzumab Vedotin (Polivy)	CD79b	Polatuzumab	IgG1κ	mc–VC–PABC	MMAE	3.5	DLBCL	R/R DLBCL in adults after ≥2 prior therapies (in combo with bendamustine and rituximab) (10 June 2019)	GO29365 (Phase Ib/II)	[18]
Enfortumab Vedotin (Padcev)	Nectin-4	Enfortumab	IgG1κ	mc–VC–PABC	MMAE	3.8	UC	Locally a/mUC following PD-1/L1 inhibitor and platinum chemotherapy, or in cisplatin-ineligible patients with prior therapy (18 December 2019)	EV-201 (Phase II)	[19]
Trastuzumab Deruxtecan (Enhertu)	HER2	Trastuzumab	IgG1κ	mc-Gly-Gly-Phe-Gly	DXd	7–8	BC	Unresectable or metastatic HER2+ BC in adults who have received prior anti-HER2 therapy in the metastatic setting, or in the neoadjuvant/adjuvant setting with disease recurrence during or within 6 months of completing therapy (20 December 2019)	DESTINY-Breast01 (Phase II)	[20]
								Unresectable or metastatic HER2-low (IHC 1+ or 2+/ISH-) BC in adults who have received prior chemotherapy in the metastatic setting, or experienced disease recurrence during or within 6 months of completing adjuvant chemotherapy (5 August 2022)	DESTINY-Breast04 (Phase III)	[21]
								Unresectable or metastatic HR+ BC with HER2-low (IHC 1+ or 2+/ISH-) or HER2-ultralow (IHC 0 with membrane staining) expression, in patients whose disease has progressed on one or more endocrine therapies in the metastatic setting (27 January 2025)	DESTINY-Breast06 (Phase III)	[22]
							Gastric or GEJ adenocarcinoma	Locally a/m HER2-positive gastric or GEJ adenocarcinoma in adults who have received a prior trastuzumab-based regimen (15 January 2021)	DESTINY-Gastric01 (Phase II)	[23]
							NSCLC	Unresectable or metastatic in adults with activating HER2 (ERBB2) mutations, as identified by an FDA-approved test, who have received prior systemic therapy (11 August 2022)	DESTINY-Lung02 (Phase II)	[24]
							Solid tumors	Unresectable or metastatic HER2+ (IHC 3+) solid tumors in adults who have received prior systemic treatment and have no satisfactory alternative treatment options (5 April 2024)	DESTINY-PanTumor02, DESTINY-Lung01, DESTINY-CRC02 (Phase II)	[25,26,27]
Sacituzumab Govitecan (Trodelvy)	TROP-2	Sacituzumab	IgG1κ	CL2 A	SN-38	7.6	BC	Unresectable locally a/m TNBC in patients who have received at least two prior systemic therapies, including at least one for metastatic disease (22 April 2020)	IMMU-132-01 (Phase I/II)	[28]
								Unresectable locally a/m HR+, HER2- BC in patients who have received prior endocrine therapy and at least two additional systemic therapies in the metastatic setting (3 February 2023)	TROPiCS-02 (Phase III)	[29]
							mUC	Locally a/m UC who previously received a platinum-containing chemotherapy and either a PD-1 or a PD-L1 inhibitor (13 April 2021; Withdrawn: November 2024)	TROPHY (Phase II)	[30]
Belantamab Mafodotin (Belamaf)	BCMA	Benlantamab	IgG1	Maleimidocaproyl	MMAF	4	MM	R/R multiple myeloma in adults after ≥4 prior therapies, including an anti-CD38 monoclonal antibody, a proteasome inhibitor, and an immunomodulatory agent (5 August 2020, Withdrawn February 2023)	DREAMM-2 (Phase II)	[31]
								With bortezomib and dexamethasone for adults with R/R multiple myeloma who have received at least two prior lines of therapy, including a proteasome inhibitor and an immunomodulatory agent (23 October 2025)	DREAMM-7 (Phase III)	
Loncastuximab Tesirine (Zynlonta)	CD19	Loncastuximab	IgG1κ	PEG-Val-Ala- PABC	PBD SG3199	2.3	DLBCL	R/R large B-cell lymphoma in adults after ≥2 prior systemic therapies, including DLBCL not otherwise specified, DLBCL arising from low-grade lymphoma, and high-grade B-cell lymphoma (23 April 2021)	LOTIS-2 (Phase II)	[32]
Tisotumab Vedotin (Tivdak)	TF	Tisotumab	IgG1κ	mc-VC–PABC	MMAE	4	CC	Recurrent or metastatic cervical cancer with disease progression on or after chemotherapy (20 September 2021)	innovaTV 204 (Phase II)	[33]
Mirvetuximab Soravtansine (Elahere)	FRα	Mirvetuximab	IgG1κ	Sulfo-SPDB	DM4	3.3–5	Epithelial ovarian cancer, Fallopian tube cancer, Peritoneal cancer	FRα-positive, platinum-resistant epithelial ovarian, fallopian tube, or primary peritoneal cancer in adults who have received one to three prior systemic therapies (14 November 2022)	SORAYA (Phase II)	[34]
Datopotamab Deruxtecan (Datroway)	TROP-2	Datopotamab	IgG1	mc-Gly-Gly-Phe-Gly	DXd	4	BC	Unresectable or metastatic HR+, HER2- (IHC 0, 1+, or 2+/ISH-) BC in adults who have received prior endocrine-based therapy and chemotherapy in the unresectable or metastatic setting (17 January 2025)	TROPION-Breast01 (Phase III)	[35,36]
							NSCLC	Locally a/m EGFR-mutated NSCLC in adults who have received prior EGFR-targeted therapy and platinum-based chemotherapy (23 June 2025)	TROPION-Lung05 (Phase II), TROPION-Lung01 (Phase III)	[35,37,38]
Telisotuzumab Vedotin (Emrelis)	c-Met	Telisotuzumab	IgG1κ	mc–VC–PABC	MMAE	3.1	NSCLC	Locally a/m non-squamous NSCLC with high c-Met protein overexpression (≥50% of tumor cells with strong [3+] staining), as determined by an FDA-approved test, in adults who have received prior systemic therapy (14 May 2025)	LUMINOSITY (Phase II)	[39]

DAR: drug-to-antibody ratio, a/m: advanced or metastatic, ALCL: anaplastic large-cell lymphoma, ALL: acute lymphoblastic leukemia, AML: acute myeloid leukemia, ASCT: autologous stem cell transplant, BC: breast cancer, CC: cervical cancer, CD22+: CD22-positive, CD33+: CD33-positive, DLBCL: diffuse large B-cell lymphoma, EBC: early stage breast cancer, GEJ: gastroesophageal junction, HCL: hairy cell leukemia, HER2-: HER2-negative, HER2+: HER2-positive, HL: Hodgkin lymphoma, mBC: metastatic breast cancer, MF: mycosis fungoides, MM: multiple myeloma, mUC: metastatic urothelial carcinoma, NSCLC: non-small-cell lung cancer, PD-1: programmed death receptor-1, PD-L1: programmed death-ligand 1, pcALCL: primary cutaneous anaplastic large-cell lymphoma, R/R: relapsed or refractory, sALCL: systemic anaplastic large-cell lymphoma, TNBC: triple-negative breast cancer, UC: urothelial carcinoma.

## Data Availability

Data sharing not applicable.

## Abbreviations

The following abbreviations are used in this manuscript:

5-FU	5-fluorouracil
a/m	advanced or metastatic
a/mBC	advanced or metastatic breast cancer
A + AVD	brentuximab vedotin, doxorubicin, vinblastine, and dacarbazine
ABVD	doxorubicin, bleomycin, vinblastine, and dacarbazine
ADCs	antibody–drug conjugates
AGAs	actionable genomic alterations
ALL	acute lymphoblastic leukemia
AML	acute myeloid leukemia
AN + AD	brentuximab vedotin, nivolumab, doxorubicin, and dacarbazine
B-ALL	B-cell precursor acute lymphoblastic leukemia
B-NHL	B-cell non-Hodgkin lymphoma
BCBM	breast cancer brain metastases
BICR	blinded independent central review
CAPE	capecitabine
CAYA	children, adolescents, and young adults
cHL	classical Hodgkin lymphoma
CMR	complete molecular response
cORR	confirmed objective response rate
CR	complete response
DA-EPOCH-R	dose-adjusted etoposide, prednisone, vincristine, cyclophosphamide, doxorubicin, and rituximab
DAS	dasatinib
Dato-DXd	datopotamab deruxtecan
DDR	DNA damage response
DFS	disease-free survival
DHL/THL	double-hit and triple-hit lymphomas
DLBCL	diffuse large B-cell lymphoma
DLTs	dose-limiting toxicities
DOR	duration of response
EBC	early breast cancer
EC	endometrial cancer
EFS	event-free survival
ENKTL	extranodal NK/T-cell lymphoma
EOC	epithelial ovarian cancer
EOT	end of treatment
FDA	the United States Food and Drug Administration
FL	follicular lymphoma
FRα	folate receptor alpha
Gas	genomic alterations
GC	gastric cancer
GCB	germinal center B-cell-like
GCTs	germ cell tumors
GEA/GEJC	gastroesophageal adenocarcinoma/gastroesophageal junction cancer
GEJ	gastroesophageal junction
GO	gemtuzumab ozogamicin
GU	genitourinary
HER2+	HER2-positive
HER2e	HER2 overexpression
HER2m	HER2 mutations
HL	Hodgkin lymphoma
HR	hazard ratio
IBC	inflammatory breast cancer
ICIs	immune checkpoint inhibitors
ICR	independent central review
iDFS	invasive disease-free survival
InO	inotuzumab ozogamicin
IRC	Independent Review Committee
IV	intravenous
la/mBC	locally advanced or metastatic breast cancer
la/mUC	locally advanced or metastatic urothelial carcinoma
LBCL	large B-cell lymphoma
Len	lenalidomide
LT	loncastuximab tesirine
M-Pola	mosunetuzumab SC and polatuzumab vedotin
mAb	monoclonal antibody
mBC	metastatic breast cancer
mDOR	median duration of response
mGEAC	metastatic gastroesophageal adenocarcinoma
mini-Hyper-CVD	dose-reduced cyclophosphamide, vincristine, and dexamethasone
MIRV	mirvetuximab soravtansine
MMR	major molecular response
mNSCLC	metastatic NSCLC
mPFS	median progression-free survival
MRD	minimal residual disease
MTD	maximum tolerated dose
mUC	metastatic urothelial carcinoma
ND	newly diagnosed
NGS	next-generation sequencing
NHL	non-Hodgkin lymphoma
NR	not reached
NSCLC	non-small-cell lung cancer
ORR	objective response rates
OS	overall survival
pCR	pathologic complete response
PD-1	programmed cell Death protein-1
PDE4	phosphodiesterase-4
PFS	progression-free survival
Ph	Philadelphia chromosome
PMBL	primary mediastinal large B-cell lymphoma
Pola	polatuzumab vedotin
Pola-R-CHP	Pola, rituximab, cyclophosphamide, doxorubicin, and prednisone
Pola-ZR	Pola, zanubrutinib, and rituximab
Pola-BR	Pola, bendamustine and rituximab
POMP	6-mercaptopurine, vincristine, methotrexate, and prednisone
PR	partial response
PROC	platinum-resistant ovarian cancer
PSOC	platinum-sensitive ovarian cancer
PTCL	peripheral T-cell lymphoma
R-CHOP	rituximab, cyclophosphamide, doxorubicin, vincristine, and prednisone
R-CHP	rituximab, cyclophosphamide, doxorubicin, and prednisone
R-GemOx	rituximab, gemcitabine, and oxaliplatin
r/mCC	recurrent or metastatic cervical cancer
R/R	relapsed or refractory
R	rituximab
RANO-BM	response assessment in neuro-oncology-brain metastases
RDE	recommended dose for expansion
RFS	relapse-free survival
RP2D	recommended phase II dose
SC	subcutaneous
SCT	stem cell transplantation
SOS	sinusoidal obstruction syndrome
T-DM1	trastuzumab emtansine
T-DXd	trastuzumab deruxtecan
Teliso-V	telisotuzumab vedotin
TNBC	triple-negative breast cancer
Ven	venetoclax
